# Pathobiology of the autophagy-lysosomal pathway in the Huntington’s disease brain

**DOI:** 10.1186/s40478-025-02131-8

**Published:** 2025-11-07

**Authors:** Martin J. Berg, Veeranna, Corrinne M. Rosa, Asok Kumar, Panaiyur S. Mohan, Philip Stavrides, Sandipkumar Darji, Deanna M. Marchionini, Dun-Sheng Yang, Ralph A. Nixon

**Affiliations:** 1 Center for Dementia Research, Nathan S. Kline Institute, 140 Old Orangeburg Road, Orangeburg, NY 10962 USA; 2 Department of Psychiatry, New York University Grossman School of Medicine, New York, NY 10016 USA; 3 Department of Cell Biology, New York University Grossman School of Medicine, New York, NY 10016 USA; 4 NYU Neuroscience Institute, New York University Grossman School of Medicine, New York, NY 10016 USA; 5 CHDI Management Inc., the company that manages the scientific activities of CHDI Foundation, Inc., New York, NY 10001 USA

**Keywords:** Huntington’s disease, Autophagy, Lysosome, Human brain, Pathobiology

## Abstract

**Background:**

Accumulated levels of mutant huntingtin protein (mHTT) and its fragments are considered contributors to the pathogenesis of Huntington’s disease (HD). Stimulating autophagy may enhance clearance of mHTT and its aggregates which has been considered as a possible therapeutic strategy. However, the role and competence of the autophagy-lysosomal pathway (ALP) during HD progression in the human disease remains largely unknown.

**Methods:**

Here, we used multiplex confocal and ultrastructural immunocytochemical analyses of ALP functional markers in relation to mHTT aggresome pathology in striatum and the less affected cortex or cerebellum of HD brains staged from Grade HD2 to HD4 by Vonsattel neuropathological criteria compared to controls.

**Results:**

Immunolabeling revealed the localization of HTT/mHTT in ALP vesicular compartments labeled by autophagy-related adaptor proteins sequestosome 1 (p62/SQSTM1) and ubiquitin, and cathepsin D (CTSD) as well as HTT-positive inclusions. Although comparatively normal at HD2, neurons at later HD stages exhibited progressive enlargement and clustering of CTSD-immunoreactive autolysosomes/lysosomes and, ultrastructurally, autophagic vacuole/lipofuscin granules accumulated progressively, more prominently in striatum than cortex. These changes were accompanied by rises in levels of HTT/mHTT and p62/SQSTM1, particularly their fragments, in striatum but not in the cortex, and by increases of LAMP1 and LAMP2 RNA and LAMP1 protein. In addition, cargo-loaded autophagosomes and cathepsin-positive autolysosomes were readily observed, implying a lack of significant blockage in autophagosome formation and autophagosome-lysosome fusion.

**Conclusions:**

The findings collectively suggest that upregulated lysosomal biogenesis and preserved proteolysis maintain autophagic clearance in early-stage HD, but the observed progressive HTT build-up and AL accumulation at advanced disease stages may signify a failure in autophagy substrate clearance. These findings support the prospect that ALP stimulation applied at early disease stages, when clearance machinery is fully competent, could lead to therapeutic benefits in HD patients.

**Supplementary Information:**

The online version contains supplementary material available at 10.1186/s40478-025-02131-8.

## Introduction

Huntington’s disease (HD) is caused by a mutation in the gene encoding the huntingtin protein (HTT) resulting in expansion of the polyglutamine (polyQ) stretch on its amino-terminus (1–4). In the neostriatum, the brain region most vulnerable and devastated by pathology in HD, the spatiotemporal advance of atrophy dorso-ventrally, caudo-rostrally, and medio-laterally has been used to stage disease pathology severity as Vonsattel’s Grades 0–4 (HD0-HD4) (5, 6). Striatal GABA-containing medium spiny projection neurons are most susceptible to cell death, even in premanifest HD0, while other striatal neuronal populations, such as aspiny interneurons, seem more resistant to toxicity (6–8). Widespread heterogeneous loss of total neurons and pyramidal cells have also been observed across many cerebral cortical regions, especially in the more advanced disease stages (9, 10). More recent studies have revealed that loss of Layer Va pyramidal neurons, identified as corticostriatal cells, can occur in the early stages of HD (11).

As in other neurodegenerative diseases, a common theme is proteinopathy due to occurrence and build-up of aggregated proteins. In HD brains neuronal intranuclear inclusions (NII) and neuropil inclusions are present (12) and these are positive for mutant huntingtin (mHTT) and ubiquitin (Ub) (12–14), suggesting that there may be a deficiency in the proteolytic machinery responsible for normally clearing these proteins, resulting in their accumulation. Autophagy is generally the principal mechanism by which cells clear organelles, long-lived proteins and damaged, misfolded, or aggregated proteins that are poor substrates for the ubiquitin–proteasome system (UPS). Both autophagy and the proteasome are implicated in HD pathogenesis (15–17). The weight of -omic evidence in mouse model Q175 and in HD brain supports the idea that proteostasis is disturbed in HD through both UPS and ALP routes (18–20). In the case of a number of proteinopathic neurodegenerative disorders (AD, PD, HD, ALS and prion diseases), aggregates are associated with and contribute to decrease in UPS (21, 22), e.g., due to proteotoxicity (23, 24) or blockage of the proteasome entrance by aggregated proteins (25). In cell or mouse models of HD, HTT accumulates in autophagosomes (AP) and autolysosomes (AL) along with lysosomal cathepsin D (CTSD) in proportion to HTT polyQ length. This has suggested that the autophagy-lysosomal pathway (ALP) may be a significant pathway for HTT proteolysis, particularly forms that misfold and potentially aggregate (26–30).

Studies in the past decade, conducted primarily with cell and/or animal models, have demonstrated multiple roles of HTT and mHTT in autophagy in relation to HD (31–33). Wild type HTT participates in normal autophagy by (1) releasing unc-51 like autophagy activating kinase 1 (ULK1) from the inhibition of the mechanistic target of rapamycin kinase (mTOR) and (2) serving as a scaffold to facilitate cargo sequestration through improving the interaction of Sequestosome 1 (SQSTM1, p62) with ubiquitinated cargos and with microtubule-associated protein 1 light chain 3 (LC3) (34, 35). mHTT influences autophagy in HD settings in multiple ways. Although mHTT may activate autophagy by sequestering mTOR and thereby reducing mTOR activity (36), most reported mHTT effects on the ALP appear to be inhibitory for autophagy, impairing earlier stages such as initiation signaling, phagophore nucleation and cargo recognition/AP formation. These additional reported mechanisms include: binding to Rheb and promoting mTOR signaling (37); interfering with ULK1 activities leading to impairment of the BECN1-PIK3C3/VPS34 and ATG14 complex (38); impairing autophagosomal cargo recognition (35, 39); and interfering with the interaction between Ataxin 3 and BECN-1, resulting in BECN-1 degradation by the UPS (40). Additional reported effects of mHTT on the endo-lysosomal system include inducing extensive endosomal tubulation (29), reducing exocytosis and promoting AL accumulation (41), and decreasing transport of late autophagic structures from the neurites to the soma (42).

It should be noted, however, that most of the above findings have been obtained from cell and/or mouse models of HD, including recent studies using induced neurons through reprogramming human fibroblasts (42, 43). By contrast, there is a relative paucity of studies that evaluate potential alterations of the ALP in the human HD brain as compared with Alzheimer’s disease (AD) (44–47) (see below). Furthermore, investigations themselves are limited in scope, often focusing on evaluations of one or a few components of interest. For example, early studies show that activities of a small cohort of lysosomal enzymes (β-glucuronidase, α-glucosidase, dipeptidyl aminopeptidase II and CTSH) are altered in brains of patients with HD (48, 49). Fragmentary information on early neuropathological characterization of HD brain has suggested an association of HTT with endo-lysosomal compartments such as multivesicular bodies (50), and an increased frequency of dystrophic neurites (51, 52) although the extent and the specific nature and composition of dystrophic neurites in HD brain are little known.

Thus, in the HD brain, it is as yet unknown whether autophagy induction is stimulated or suppressed in neurons at any stage of the disease. It is also not known whether autophagy clearance steps, such as AP-lysosomes (LY) fusion and autolysosomal proteolytic function are competent. Such information, particularly defining the site(s) at which autophagy may be disrupted, is crucial to designing possible interventions for HD based on autophagy modulation. This laboratory has focused over a number of years on evaluating roles for autophagy in AD, as well as in Parkinson’s disease (PD) and has pioneered multimodal analysis of the many aspects of autophagy to discern where perturbation has interfered with proteostasis in authentic human disease (44–47). Therefore, in this study, we aimed to characterize the status of the ALP in relation to disease progression over the course of pathology in human brain samples, employing ultrastructural, immunohistochemical, and molecular analyses for the caudate nucleus of the striatum (STR), the prefrontal cortex (CTX) and cerebellum (CBM) from control cases and HD2-HD4 patients. To our knowledge, this study is unique in terms of the relatively large number of HD cases used (Table 1), the level of ultrastructural analysis, the range of autophagy related processes analyzed, and the value of human brain-derived information regarding this disease. In this regard, we also accessed a postmortem HD brain with exceptionally well-preserved ultrastructural preservation, enabling detailed neuropathological and immunochemical analyses of autophagy-related alterations.

**Table 1:** Demographics of human brain samples utilized for qPCR for mRNA analysis (qPCR), western blotting (WB), cathepsin enzymatic activity assay (Enzyme), immunohistochemistry (IHC) and electronic microscopy (EM)

Case no	HD Stage	PM (hr)	Age (y)	Brain banks	Analysis performed (regions used)													
					qPCR			WB			Enzyme			IHC			EM	
					STR	CTX	CBM	STR	CTX	CBM	STR	CTX	CBM	STR	CTX	CBM	STR	CTX
B7010	CTL	18.9	64	HBTRC					X			X						
B7360	CTL	38.1	51	HBTRC					X			X						
B7970	CTL	25.8	70	HBTRC					X			X						
6916	CTL	Na	Na	HBTRC										X				
6919	CTL	Na	Na	HBTRC										X	X			
8341	CTL	15.7	82	HBTRC	X	X	X	X	X	X	X	X	X					
8176	CTL	25.3	86	HBTRC	X	X	X	X	X	X	X	X	X					
8108	CTL	31.6	70	HBTRC	X	X	X	X	X	X	X	X	X					
AN01404	CTL	18.6	74	HBTRC					X			X						
AN01614	CTL	18.7	80	HBTRC					X			X						
AN03398	CTL	12.1	75	HBTRC					X			X						
AN07810	CTL	18.1	65	HBTRC					X			X						
AN11537	CTL	12.5	60	HBTRC					X			X						
AN12699	CTL	11.0	55	HBTRC					X			X						
E04-32	CTL		70	ECND										X				
E04-34	CTL	17	57	ECND	X	X	X				X	X	X	X				
E04-46	CTL	31	40	ECND	X	X	X	X		X	X		X					
E05-74	CTL	6	59	ECND	X	X	X	X	X	X	X		X	X				
E06-41	CTL	10	57	ECND	X	X	X	X	X	X	X	X	X	X				
E06-45	CTL	6.5	46	ECND	X			X		X		X	X					
E06-114	CTL	6.5	53	ECND	X	X	X	X	X	X	X	X	X		X			
E09-170	CTL	14.5	88	ECND										X				
E11-33	CTL	15	43	ECND	X		X					X		X	X			
OS03-299	CTL	6.0	69	ECND										X				
OS03-380	CTL	12	61	ECND	X	X	X				X	X						
OS03-390	CTL	7	74	ECND										X	X			
T-272	CTL	Na	Na	NYBBC							X							
T-346	CTL	10	84	NYBBC					X			X						
Avg/Tot		**16.2 +/- 8.8**	**65.3 +/- 13.4**															
E05-119	HD2	11	56	ECND	X	X	X	X	X	X	X	X	X	X	X			
E05-154	HD2	33.0	67	ECND										X				
E10-05	HD2	8.5	62	ECND	X	X	X	X	X	X	X	X	X	X	X			
OS01-04	HD2	24	51	ECND	X	X	X	X		X	X	X	X	X	X		X	X
OS01-12	HD2	na	73	ECND										X				
OS01-114	HD2	2.5	68	ECND	X	X	X	X	X	X	X	X	X	X	X	X		
T-197	HD2	2.9	78	NYBBC					X									
T-264	HD2	7.5	69	NYBBC					X									
T-272	HD2	17	83	NYBBC				X	X	X		X						
T-289	HD2	24.5	54	NYBBC					X									
T-309	HD2	4	80	NYBBC					X									
T-550	HD2		82	NYBBC	X	X		X	X	X	X	X						
T1146	HD2	39	69	NYBBC		X	X	X	X	X	X	X			X			
Avg/Tot		**15.8+/-12.7**	**68.6+/-10.6**															
B8001	HD3	18.3	47	HBTRC					X					X	X			
B8007	HD3	22.6	65	HBTRC					X					X	X		X	X
B7901	HD3	23.3	73	HBTRC					X									
7684	HD3	22.7	61	HBTRC										X	X			
7939	HD3	22.6	70	HBTRC										X	X			
7989	HD3	24	52	HBTRC										X	X		X	X
8000	HD3	15.6	59	HBTRC										X	X		X	X
8232	HD3	15.3	58	HBTRC	X	X	X	X	X	X	X	X	X					
8234	HD3	20.8	68	HBTRC	X	X	X	X	X	X	X	X	X					
8268	HD3	19.9	55	HBTRC	X	X	X	X	X	X	X	X	X					
E05-24	HD3	18.5	63	ECND	X	X	X	X	X	X	X	X	X	X	X		X	X
E05-38	HD3	12.5	70	ECND										X				
E07-188	HD3	12.5	63	ECND	X	X	X	X	X	X	X	X	X	X	X		X	X
E09-06	HD3		55	ECND										X	X		X	X
OS00-03	HD3	6.5	54	ECND	X	X	X	X	X	X	X	X	X					
OS00-09	HD3	96	64	ECND										X				
OS01-88	HD3	5	43	ECND					X					X	X			
OS99-17	HD3	8	83	ECND	X	X	X	X	X	X	X	X	X	X				
OS99-19	HD3	6.5	67	ECND										X				
T-4276	HD3	25	59	NYBBC	X	X	X	X	X	X	X	X	X					
T4584	HD3	Na	Na	NYBBC								X						
T-4830	HD3	24	63	NYBBC							X	X	X					
Avg/Tot		**21.0 +/- 18. 8**	61.5 +/- **9.1**															
B7624	HD4	24.1	50	HBTRC					X					X	X			
B7735	HD4	23.1	60	HBTRC					X					X	X			
B7822	HD4	29.1	54	HBTRC					X					X	X	X	X	
7319	HD4	13.5	76	HBTRC										X	X			
7681	HD4	26	74	HBTRC										X	X			
7684	HD4	Na	Na	HBTRC										X				
7791	HD4	Na	Na	HBTRC											X			
7792	HD4	13.0	42	HBTRC										X	X	X	X	
7822	HD4	29.1	54	HBTRC										X	X	X		
7939	HD4	24	52	HBTRC										X				
7989	HD4	Na	Na	HBTRC										X				
7991	HD4	21.8	71	HBTRC	X	X	X	X	X	X	X	X	X	X	X			
8083	HD4	20.1	52	HBTRC	X	X	X	X	X	X	X	X	X					
8093	HD4	24.2	51	HBTRC	X	X	X	X	X	X	X	X	X					
8150	HD4	21.1	57	HBTRC	X	X	X	X	X	X	X	X	X					
8207	HD4	29.1	80	HBTRC	X	X	X	X	X	X	X	X	X					
T-128	HD4	3.9	50	NYBBC		X												
T-141	HD4	6.4	77	NYBBC					X									
T-225	HD4	17.9	72	NYBBC					X									
T-295	HD4	1.5	46	NYBBC					X									
T-329	HD4	0.3	54	NYBBC					X									
T-4584	HD4	Na	Na	NYBBC								X						
T-4817	HD4	31.5	42	NYBBC	X	X	X				X	X	X					
T-5017	HD4	12.0	66	NYBBC												X	X	
OS01-03	HD4	5	58	ECND	X	X	X	X	X	X	X	X	X			X		
Autopsy 49	HD4	Na	Na	MSNBBR C														
1719	HD4	Na	Na	MSNBBR C												X	X	
Avg/Tot		**17.9 + 9.9**	**58.6 + 12.0**															

The significance of the bold font in the cells corresponding to the averages +/- SD for “PMI” and “Age at Death” were to highlightthe overall similarities in age matching and sample state for all cohorts used

HBTRC, Harvard Brain Tissue Resource Center; ECND, Emory Center for Neurodegenerative Disease; NYBBC, New York Brain Bank at Columbia; MSNBBRC, Mount Sinai Neuropathology Brain Bank & Research CoRE; Na, Not Available; Str, Striatum; Ctx, Cortex, BA9/10; Cbm, Cerebellum

## Results

### HTT inclusions: types and close relationships with autophagy adaptor proteins Ub and p62

**Fig. 1:**
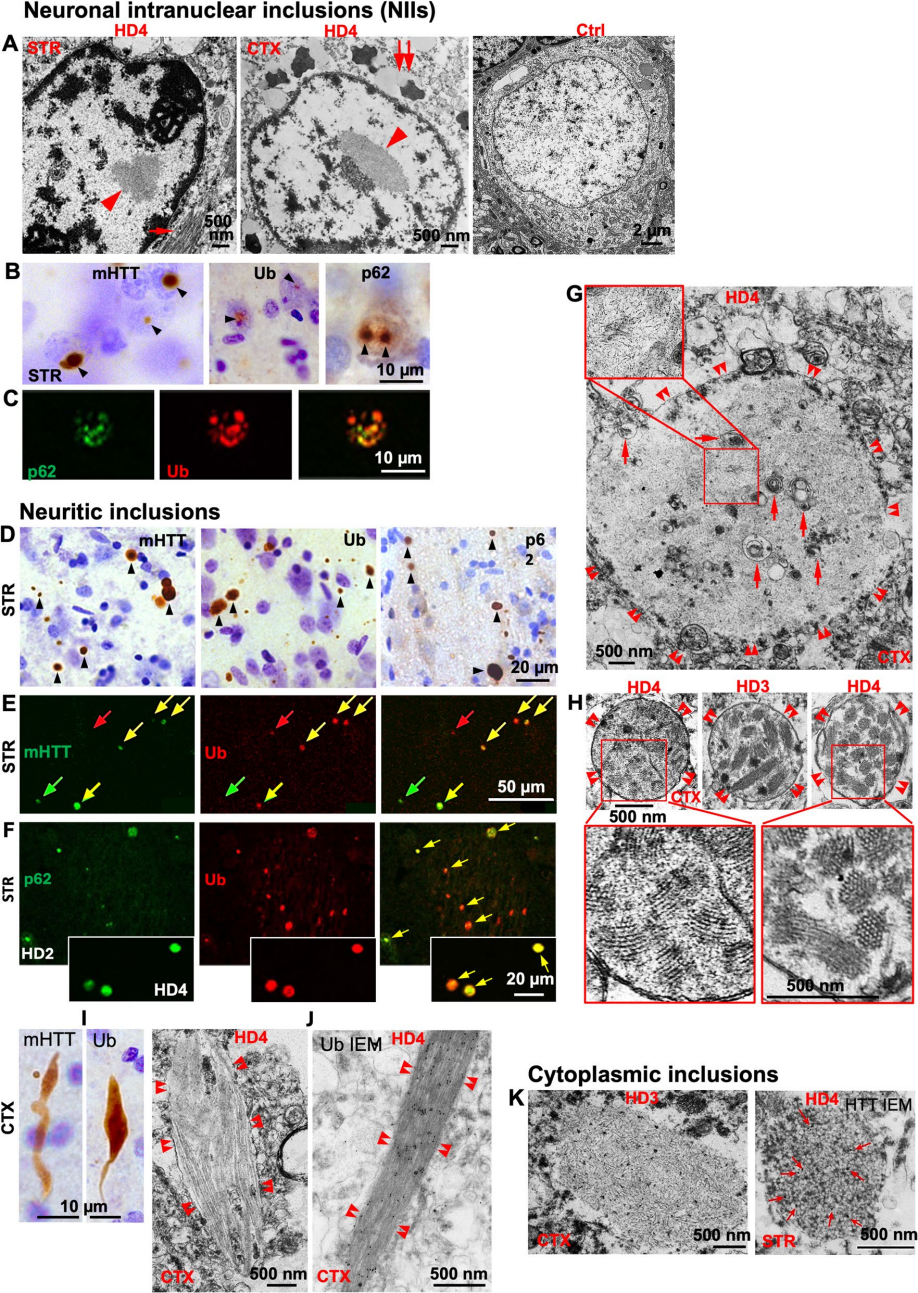
HTT inclusions: types, distributions and close relationship with autophagy adaptors Ub and p62. **A**–**C** Representative micrographs taken from the STR and/or CTX (as depicted on the individual panels) of HD brains for demonstrating NIIs. (A) EM images depicting the NII (arrowhead) in the STR (left panel) and CTX (middle panel) of a HD4 case which is absent in the control brain (right panel – high resolution image for this panel is presented as Fig. S1a.). Bar = 500 nm (left, middle) or 2 µm (right). (B) LM images showing that NIIs (arrowheads) are detected by antibodies to mHTT (mEM48), pan-Ub or p62. Bar = 10 µm. (C) Confocal images from brain sections double-labeled with anti-p62 and -Ub antibodies depicting a high degree of colocalization of the two markers within NIIs. Bar = 10 µm. **D**–**J** Representative micrographs taken from the STR and/or CTX (as depicted on the individual panels) of HD brains for demonstrating neuritic inclusions. (D) LM images showing neuritic inclusions (arrowheads) in the neuropil detected by antibodies to mHTT (mEM48), pan-Ub or p62. Bar = 20 µm. (E, F) Confocal images from brain sections double-labeled with anti-mHTT (mEM48) and -pan-Ub (E), or anti-p62 and -pan-Ub (F) antibodies demonstrating labeled neuritic inclusions (arrows) in the neuropil. Yellow arrows depict double-labeled inclusions indicating colocalization, while red and green arrows point to singly labeled inclusions without colocalization. Bar = 50 µm (E, F) and = 20 µm (F, insets). (G) A representative EM image for the most common type (see text) of neuritic inclusions (double arrowheads depict the boundary of the neurite), usually 1–8 µm in diameter, filled with short, fine fibrous elements (Inset). Arrows indicate AV within the inclusions. Bar = 500 nm. (H) Representative EM images for the less common type (see text) of neuritic inclusions (circled by double arrowheads), usually 0.5–2 µm in diameter, characterized by a fingerprint feature, appearing to be composed of bundles of filaments/microtubules. Bar = 500 nm. (I, J) Representative LM (I) and EM images (J) for the least common type (see text) of neuritic inclusions (surrounded by double arrowheads). (I) shows the elongated feature of the inclusions revealed by mHTT (mEM48) or pan-Ub antibodies. Bars = 10 µm. (J) depicts the filaments/microtubules revealed by either conventional EM or IEM with an anti-pan-Ub antibody, indicating specific immunogold labeling on the filaments. Bars = 500 nm. **K** Representative EM micrographs taken from HD brains for demonstrating cytoplasmic inclusions, which exhibit as either accumulation of fibrous filaments devoid of a limiting membrane (K, left), or accumulation of very small (< 30 nm in diameter) clear vesicles which are positive for mHTT (mEM48) as shown by labeling of immunogold particles (red arrows) (K, right). High resolution images for (K) are presented as Fig. S1d

Consistent with previous studies reporting NIIs in human HD brains (12), our ultrastructural analysis of affected neurons in the STR and CTX revealed nuclei containing single discrete ovoid or irregular shaped NIIs, 1–4 µm in diameter, composed of a relatively uniform meshwork of granular or short fibrous elements (Fig. 1A, left and middle, arrowheads), which were not detected in control brains (Fig. 1A, right and Fig. S1a). At the light microscope level, NIIs were readily detected by antibodies directed against HTT (mEM48) or autophagy adaptor proteins including Ub and p62 (Fig. 1B). The extent to which nuclei were filled by mHTT, Ub or p62 immunoreactivity (IR) varied, ranging from a discrete small punctum, to the rimming of the outer surface of the nuclear envelope, to finally a progressive labeling of the entire nucleus (Fig. S1b—using Ub as an example). Double immunofluorescence labeling detected a high degree of colocalization of nuclear p62-IR and Ub-IR (Fig. 1C).

Another major type of inclusion noted were neuritic inclusions that randomly distribute in the neuropil. They were also readily labelled by anti-mHTT, -Ub and -p62 antibodies both in the STR (Fig. 1D–F) and the CTX (Fig. S1c). The majority of neuritic inclusions exhibited spherical or oval shapes, with highly variable sizes (0.5–8 µm) (Fig. 1D–F; Fig. S1C). Upon double labelling and confocal immunofluorescent microscopy high levels of colocalization were observed between mHTT-IR and Ub-IR (Fig. 1E) or p62-IR and Ub-IR (Fig. 1F), implying colocalization of the three proteins within the inclusions, a pattern replicated in our study in the brain of a knock-in HD mouse model Q175 (53). The extent of overlapped area of the two colors from p62-IR and Ub-IR appeared to increase with disease progression (HD2 in Fig. 1F vs HD4 in Fig. 1F insets), suggesting that p62 and Ub proteins, and mHTT as well, each can aggregate independently earlier and then further develop to form composite structures. Besides these spherical/ovoid neuritic inclusions, there were elongated types of modestly enlarged neurites (Fig. 1I) with much lower frequency – for example, they were hardly found even in images at low magnification (Fig. 1D; Fig. S1C).

Ultrastructural analysis highlights three types of inclusions found within the neuropil. (1) The most common of these were 1–8 µm spherical inclusions with no apparent limiting membrane. Many of these occupied the entire cross-sectional area of a given neurite, leaving only a narrow surround of cytoplasm between the inclusion and the plasma membrane of the neurite (Fig. 1G). The internal structure of these inclusions, like those in the nucleus, consisted of short, fine fibrous elements (Fig. 1G, Inset). Autophagic vacuoles (AV) or other small membranous vesicles were sometimes trapped within these structures (Fig. 1G, arrows). (2) The second type of inclusion in the neuropil, which were slightly less common than the above inclusions, were 0.5–2.0 µm membrane-bound spherical structures containing multiple smaller fingerprint profiles, most of which appeared to be composed of well-arranged bundles of filaments or microtubules (Fig. 1H). These two forms of inclusions may correspond to the spherical/ovoid type found at the light microscopic level (Fig. 1D–F). (3) The third and least common inclusions in the neuropil were elongated or comet-shaped structures (Fig. 1J), most likely corresponding to the aforementioned elongated type detected under light microscopy with DAB-immunolabelling (Fig. 1I), which were partially or fully occupied by microtubule-like elements (Fig. 1J, left) or fibrillar bundles which were positive for Ub as detected by Immuno-Gold EM (IEM) (Fig. 1J, right).

In addition to the NIIs and the neuritic inclusions, there were cytoplasmic inclusions, which, using EM, could be identified as 3 forms. (1) one or several large (~ 3 µm) ovoid or spherical structures devoid of limiting membrane and consisting of mainly a meshwork of short, thin fibrous elements intermixed with small numbers of membranous elements (Fig. 1K, left; Fig. S1D). These inclusions resembled the similar size fibrous structures seen in the neuropil (Fig. 1G). (2) 2–5 µm membrane-bound profiles containing collections of very small clear vesicles (< 30 nm diameter) (Fig. 1K, right; Fig. S1D). (3) Fiber bundles similar to those found in the neuropil (e.g., Fig. 1J) were occasionally observed perinuclearly within the cytoplasm (Fig. 1A, left, arrow).

## qPCR of selected ALP-related targets in HD brains

**Fig. 2:**
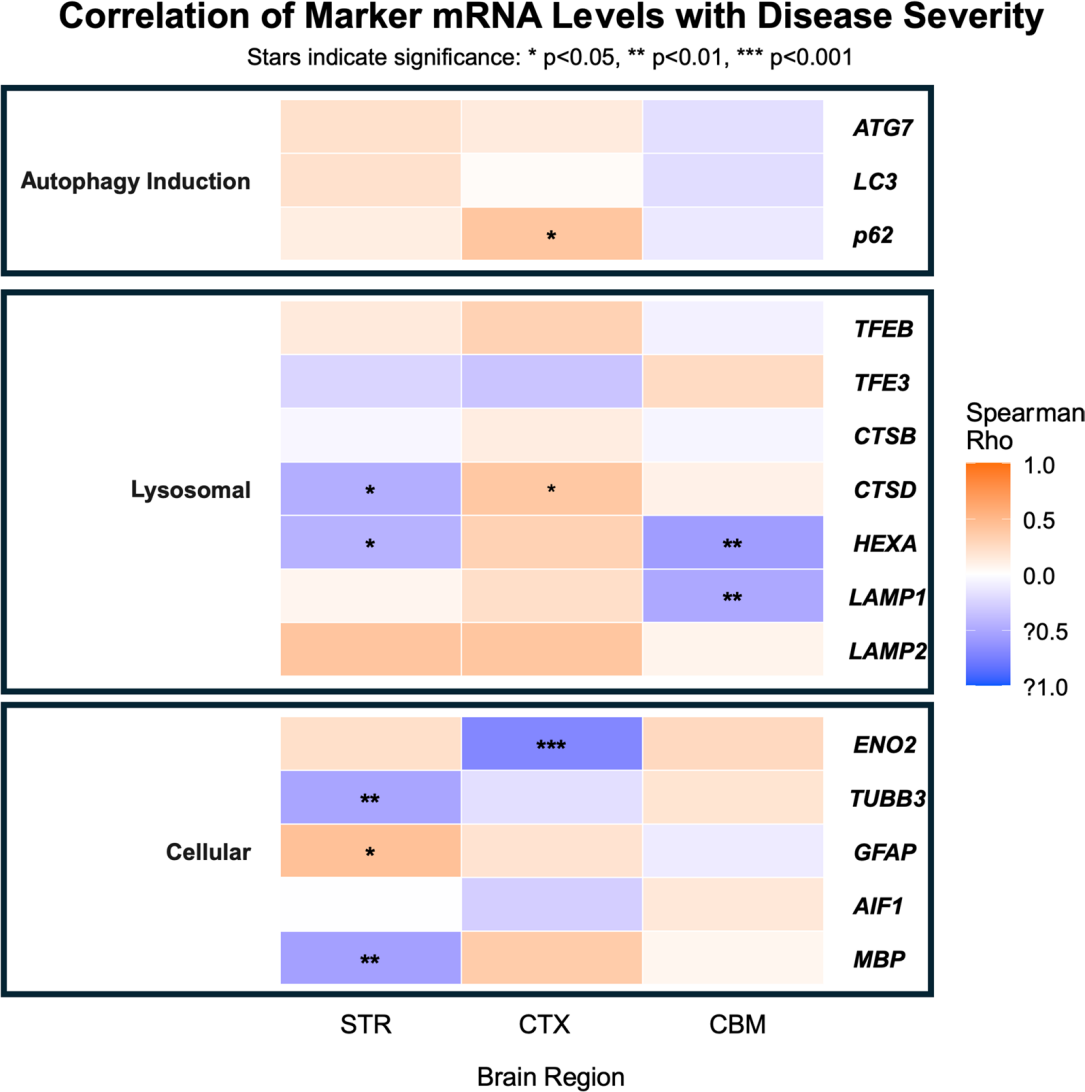
Correlation between marker expression levels and HD progression stages across different brain regions, grouped by marker category. The heatmap illustrates Spearman’s rank correlation coefficients (ρ) between the relative mRNA expression levels from qPCR of selected molecular markers and Huntington’s disease (HD) severity. Analyses were performed on human post-mortem brain tissue from control (Ctl) and HD patients (grades HD2, HD3, HD4), with severity numerically encoded for correlation (Ctl = 0, HD2 = 1, HD3 = 2, HD4 = 3). Correlations are shown for STR, CTX, and CBM). Markers are grouped into functional categories, displayed from top to bottom: Induction Markers (Autophagy induction): ATG7, LC3, p62. Lysosomal Markers (Lysosomal biogenesis, hydrolases and structural elements): TFEB, TFE3, CTSB, CTSD, HEXA, LAMP1, LAMP2. Cellular Markers (cellular processes): ENO2, TUBB3, GFAP, AIF1, MBP. The color of each cell represents the Spearman ρ value, with a continuous gradient from blue (strong negative correlation, ρ = −1) through white (no correlation, ρ = 0) to red (strong positive correlation, ρ =  + 1). Nominal p-values for the correlations are indicated by asterisks: *p < 0.05, **p < 0.01, ***p < 0.001.

We next examined transcripts of genes in the ALP with RNA samples prepared from the collected brain regions from identical samples also used subsequently in immunoblot assays (see Table 1 Demographics). qPCR targets shown in Fig. 2 come from a group of targets chosen to assay aspects of autophagy from induction, transcriptional activation and lysosomal function and structure affecting autophagic clearance and flux. These are supplemented by a cohort of marker genes to address changes in neuronal and glial populations. ΔΔCT values were then grouped by their Vonsattel staging as follows: Ctl = 0, HD2-4 = 1, 2, or 3. As shown in Fig. 2, CTX p62 (SQSTM1) expression positively correlated significantly with disease severity whereas ATG7 and LC3b expression in both CTX and STR trended upward while there was a negative correlation for these markers of autophagy induction or cargoes in CBM. On the other hand, transcriptional activators for lysosomal biogenesis performed similarly to results from AD brain with TFEB levels trending upward and TFE3 performing in the opposite, but neither reaching significance in all regions (44). Lysosomal CTSD was significantly corelated with disease in CTX but was significantly depressed in STR as was HEXA in both STR and CBM. Other hydrolases (CTSB, HEXA) and membrane proteins LAMP1/2 were trending upward as well in CTX but lacked significance. These data point to mild alterations in the latter stages of autophagy related to substrate clearance in CTX and especially STR relative to CBM that is trending downward with disease progression. In fact, STR and CTX show a negative correlation overall when compared with the CBM (data not shown). Regarding cell markers, neuron-specific ENO2 and TUBB3 stand as proxies for neuronal loss where they were negatively correlated in CTX and STR respectively, whereas both were trending upward in CBM as an area affected to a lesser extent (54). On the other hand, significantly correlated increase of GFAP in STR acts as an indicator of gliotic changes, while decrease in MBP may reflect myelin deficits (55).

## Immunoblotting analyses of HTT proteins and adaptor proteins p62, TRAF6 and Ub

**Fig. 3:**
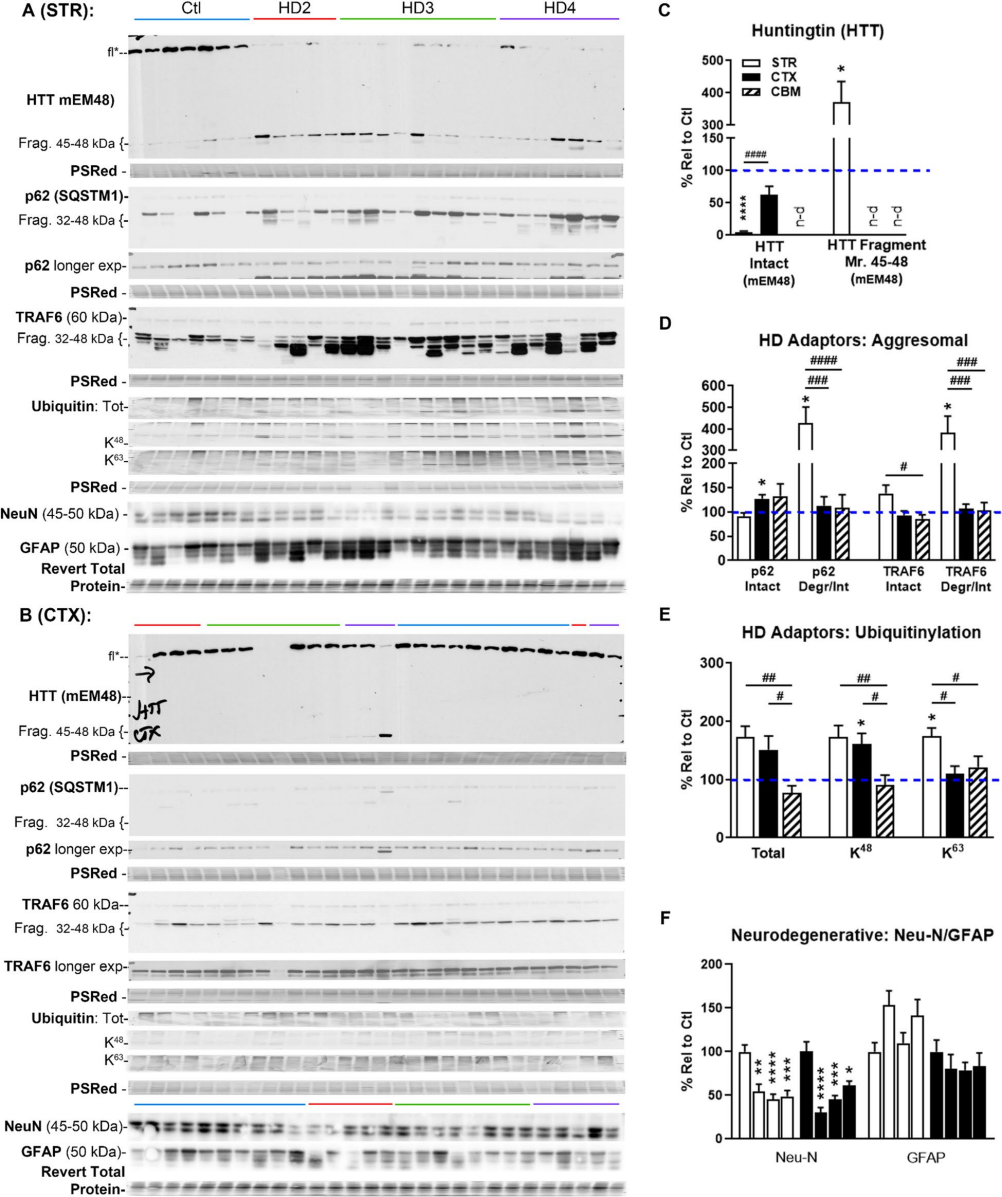
Protein levels of HTT and autophagy adaptors Ub, p62 and TRAF6 in the STR and CTX. **A**, **B** Western blots of samples run in 10% Tris–glycine gels (each lane representing a different HD patient or control case) from the STR (i.e., the caudate nucleus of the striatum) (A) and CTX (i.e., the prefrontal cortex) (B) for assessing the levels of intact and/or fragmented HTT species (with antibody mEM48), as well as UPS/autophagy adaptor proteins including p62, TRAF6 and Ub. All tissue samples (25 µg) were run at the same time in wide-format single-percentage or in precast gradient gels, electroblotted onto same membrane for immunodetection by ECL. Specific antigens were normalized and quantitated against total protein staining or using housekeeping proteins, with representative Ponceau S Red-stained blots or ACTB blots (detected by colorimetric assay using DAB) shown in the figure. Color bars above the blots denote HD staging (Blue = Control/Ctl; Red = HD2; Green = HD3; Purple = HD4). (C-E) Bar graphs showing the quantitative results for STR, CTX and CBM samples (Western blots of CBM are in Suppl. Fig. S2), obtained after normalization with the loading control, i.e., ACTB bands and/or bands on uncompressed original Ponceau S Red stained blots. Columns in C-E represent pooled HD stages (HD2-4 samples). Values are expressed as “% relative to Control” pooled values (denoted by dashed blue lines) ± SEM. The values and variability (“error bar”) of the pooled Control groups, to which the pooled HD groups are normalized, are expressed as 100% ± SEM and listed below: for intact and fragmented HTT species (intact HTT = 100 ± 24.41 STR, ± 25.8 CTX, n.d CBM; degr HTT = 100 ± 32.32 STR, n.d. CTX, n.d. CBM) (**C**), for adaptor protein/aggresomal markers (p62 intact = 100 ± 13.58 STR, ± 9.93 CTX, ± 23.96 CBM; degr p62 = 100 ± 31.40 STR, ± 16.80 CTX, ± 28.65 CBM; TRAF6 intact = 100 ± 11.03 STR, ± 18.40 CTX, ± 16.97 CBM; degr TRAF6 = 100 ± 17.25 STR, ± 11.48 CTX, ± 19.71 CBM) (**D**), for adaptor protein/ubiquitination species (Total Ub = 100 ± 28.68 STR, ± 18.07 CTX, ± 21.17 CBM; K^48^ = 100 ± 25.67 STR, ± 17.51 CTX, ± 20.05 CBM; K^63^ ± 20.71 STR, ± 12.18 CTX, ± 19.69 CBM) (**E**). (**F**) for NeuN/GFAP levels in STR and CTX (as a proxy for neurodegeneration) where the value of each Control group is shown as a separated column with their own error bar and therefore the order of columns for each brain region shown in F is Ctl, HD2/3/4 (left to right). n-d: not detectable. Significant differences assessed by ANOVA analysis with post-hoc Dunnett’s comparison to control or Tukey’s to highlight significant differences between STR and CTX columns. Symbols *: comparisons with the Ctl; # signs: comparisons among STR, CTX and CBM. * or # *P* < 0.05, ** or ## *P* < 0.01, *** or ### *P* < 0.001, **** or #### *P* < 0.0001. STR and CBM n (Ctl) = 7, n (HD2) = 5, n (HD3) = 9, n (HD4) = 7; CTX n (Ctl) = 10, n (HD2) = 5, n (HD3) = 8, n (HD4) = 5. Note that the images of blots for ubiquitination species or PSRed staining were compressed in the vertical direction to be 15% relative to the original height, but quantitation was done using full length on the original blots. PSRed = Ponceau S Red staining

The morphological observations shown above suggest close spatial relationships among mHTT and the adaptor proteins Ub and p62. We further investigated such relationships biochemically by immunoblot analyses of these proteins in the STR, CTX and CBM from HD patients (Vonsattel neuropathological stages HD2-HD4) and corresponding controls (Fig. 3). As noted above, a large number of these regional samples were derived from the same patients, and qPCR analysis revealed inter-patient variability was driving regional differences in transcriptomics within the same patient-derived samples (Table 1 and data not shown), thus lending a great deal of confidence in comparing and contrasting these cohorts. That said, we observed regionally specific patterns in the levels of intact HTT protein and HTT fragments. Only quantitation for CBM is shown in Fig. 3 as levels of HTT and adaptors were lower than those found in STR or CTX, as found in blot images omitted in Fig. 3 for clarity but shown in Suppl. Fig. S2.

Levels of intact HTT (i.e., full-length HTT) were greatly reduced in the STR of HD cases (as early as HD2) compared to controls when detected by an N-terminal antibody, mEM48 (13) (Fig. 3A, fl (full-length)*; Fig. 3C, left), while the difference in the levels of this HTT species in the CTX between the control and HD cases was only marginal (Fig. 3B, fl*; Fig. 3C, left). The decline of the intact HTT in the STR of HD stages was accompanied by a marked accumulation of 45–48 kDa N-terminal fragments of HTT (Fig. 3A; Fig. 3C, right), while such fragments were largely absent in the CTX (Fig. 3B; Fig. 3C, right) (see Discussion). Both intact HTT and fragments of HTT were undetectable in CBM samples (Suppl. Fig. S2; Fig. 3C, left and right).

Immunoblotting analyses of p62 and TRAF6, two adaptor proteins known to interact with each other, revealed that these two proteins also generated proteolytic fragments (32–48 kDa) which, interestingly, also selectively increased in the HD STR at a level of nearly fourfold over the controls (Fig. 3A, and D), similar to the selective accumulation of mHTT fragments in the HD STR described above. By contrast, there were no differences in the levels of such fragments between HD and control cases in the CTX (Fig. 3B and D) or in CBM (Suppl. Fig. S2; Fig. 3D). In addition, analysis of ubiquitination revealed a significant increase in K^48^ and K^63^ ubiquitination and a strong trend of elevation in Total ubiquitination (p = 0.06) in the STR and a significant increase only in K^48^ ubiquitination in the CTX (Fig. 3A, B and E), implying that there might be a compensatory but still impaired response of the UPS to protein aggregates, whereas CBM is largely unchanged (Suppl. Fig. S2, quantitation in Fig. 3E). Together, the generation/accumulation of proteolytic fragments of mHTT, p62 and TRAF6, and the increase in ubiquitination in the STR appear to be disease-related and brain region-selective.

Levels of neuron-specific NeuN and astrocyte marker GFAP were also assayed by western blot to use as a proxy to evaluate neuronal cell death and active gliotic changes with HD staging in STR and CTX. As shown in Fig. 3A and B, NeuN immunoreactivity appears decreased, verified by significant decrease in both STR and CTX to similar levels across all HD pathological staging (Fig. 3F), whereas GFAP levels trended up in the STR, but were relatively stable in the CTX (Fig. 3A, B, F).

## ALP pathology develops at the late stages of the disease as revealed by CTSD immunolabeling

**Fig. 4:**
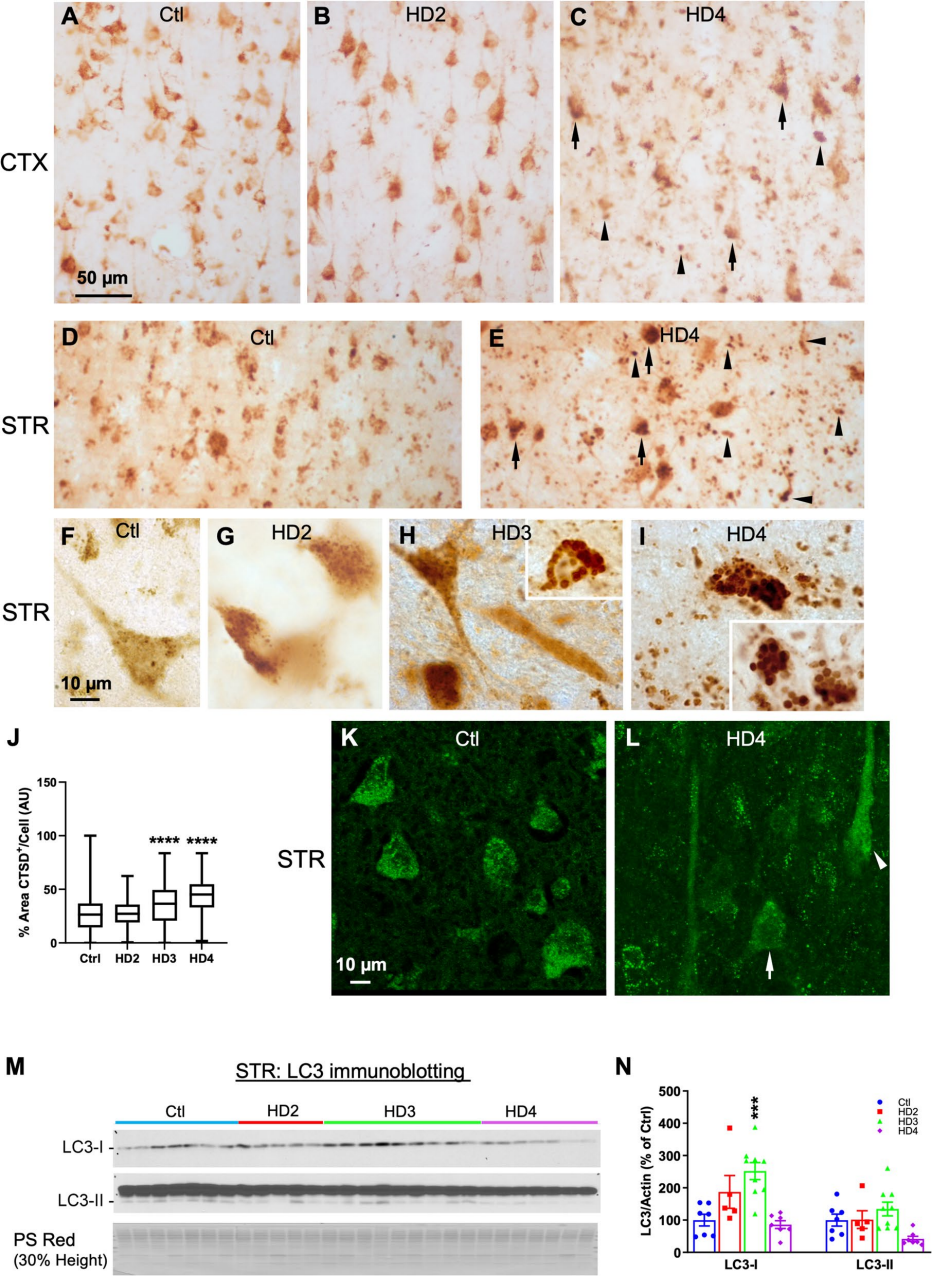
HD brains develop ALP pathology in the later stages of disease progression. **A**–**I** Brain sections from control and HD cases were immunostained with an anti-CTSD antibody. Low magnification images were taken from the CTX of Control (Ctl) (A), HD2 (B) and HD4 (C) stages and from the STR of Ctl (D) and HD4 (E): abnormal staining pattern (see text) in the HD4 brain represented by strong and clumping IR at one pole of the neurons (arrows) and strong and increased staining in swollen neurites in the neuropil (arrowheads). (F-I) High magnification images from the STR showing small punctate CTSD granules (i.e., AL/LY) in neurons of Ctl, HD2 and HD3 (F–H), while grossly enlarged positive granules present in HD3 (H, Inset) and dominantly in HD4 (I and Inset). **J** Quantitation of _ir_CTSD (%Area/Cell) in cortical pyramidal cells analyzed using the Kruskal–Wallis non-parametric test with post-hoc analysis using the Dunn’s post test with significance denoted as ****p ≤ 0.001. **K**, **L** Representative confocal images from the STR of Ctl and HD4 cases labeled for LC3. Scale bar in (A) = 50 µm for (A-E), in (F) = 10 µm for (F-I), in (K) = 10 µm for (J, K). **M** Immunoblot in 10% Tris–glycine gel for LC3 protein in the STR. Loading and analysis described in Fig. 3 legend. Each lane represents a different HD patient or control case with sample numbers shown in Fig. 3 legend. Color bars above the blots denote HD staging (Blue = Ctl; Red = HD2; Green = HD3; Purple = HD4). **N** Quantitation results following ANOVA analysis with post-hoc Dunnet’s comparison for LC3-I and -II derived from the blot shown in (M). Each bar represents normalized results for antigen relative to total protein and expressed as “% relative to Ctl” (set as 100%) ± SEM. Significant differences shown as ***p < 0.005

We subsequently assessed the status of the ALP in HD brains, compared to control brains, with immunohistochemistry (IHC) of CTSD, which captures the status of both LY and AL collectively as CTSD is a lysosomal protease existing in both organelles and can serve as a marker for the ALP (AL can be distinguished from LY by residual presence of LC3 or another autophagy-specific substrate). Anti-CTSD antibody labeled many pyramidal neurons in multiple layers of the CTX in control cases with small positive puncta (visible in some neurons even at a low magnification) (Fig. 4A). Brain sections at HD2 stage exhibited a similar CTSD labeling pattern (Fig. 4B) to that seen in the Controls, suggesting an absence of lysosomal pathology at this earlier stage of the disease progression. However, an abnormal staining pattern was observed in sections from HD4, exhibiting as stronger, clumping luminal staining in neuronal somas particularly at the basal poles, and an overall patchy staining pattern likely due to neuronal loss and increased staining in the neuropil (Fig. 4C, quantitation of cortical sections highlights increased CTSD^+^ signal with staging shown in Fig. 4J). Similarly, in the STR, an apparently normal neuronal CTSD staining pattern was seen in sections from control cases even though there was some small clumping staining (Fig. 4D), and again, abnormal staining pattern was evident at HD4 stage reflected by strongly stained puncta and clumps (of puncta) within neuronal somas or in the neuropil (Fig. 4E), which was more severe than that seen in the CTX (Fig. 4C). At a higher magnification (Fig. 4F–I), small and moderately CTSD-positive puncta, representing normal AL/LY morphology, were seen in neurons of control and HD2 cases (Fig. 4F, G). By contrast, exacerbated staining of grossly enlarged CTSD granules in neurons and neuropil became predominant at HD4 (Fig. 4I), indicating obvious lysosomal pathology. As expected, the STR at HD3 stage exhibited a CTSD staining pattern in between what is seen in HD2 and HD4, i.e., some neurons were still normal appearing (Fig. 4H), while others did become abnormal (Fig. 4H, inset). Together, lysosomal pathology, as revealed by CTSD IHC, develops to a greater extent in the STR compared with the CTX of HD brains late in the disease progression, particularly at HD3-HD4 stages.

Immunolabeling with LC3, another marker for the ALP, revealed LC3 staining (both diffuse and punctate) in neurons in the control and HD STR (Fig. 4K, L). However, even at HD4, there were no evident changes (Fig. 4L) beyond the range seen in control brains (Fig. 4K) where some neurons were labeled more strongly but diffusely (Fig. 4L, arrowhead), and others exhibited a trend of reduced staining (Fig. 4L, arrow). We further assessed the status of LC3 protein in STR samples with LC3 immunoblotting (Fig. 4M). A significant increase was detected in HD3 samples for LC3-I (Fig. 4N) whereas no differences were found for LC3-II levels (after normalizing to loading control) between Ctl and either of HD2 or HD4 even if there was a trend of LC3-II reduction in HD4 and a significant difference between HD3 and HD4 (Fig. 4N). When analyzed for the ratio of LC3-II/-I, there were no changes detected (not shown). Taken together, while the CTSD IHC was able to report defects at the later stages of the ALP, the LC3 data, including the numbers of LC3 puncta and size distribution and the levels of LC3-II, did not suggest robust alterations reflecting impairments in these particular steps of the ALP (e.g., AP formation, AP-LY fusion or AL clearance).

## Association of mHTT signal with CTSD IR during disease progression

**Fig. 5:**
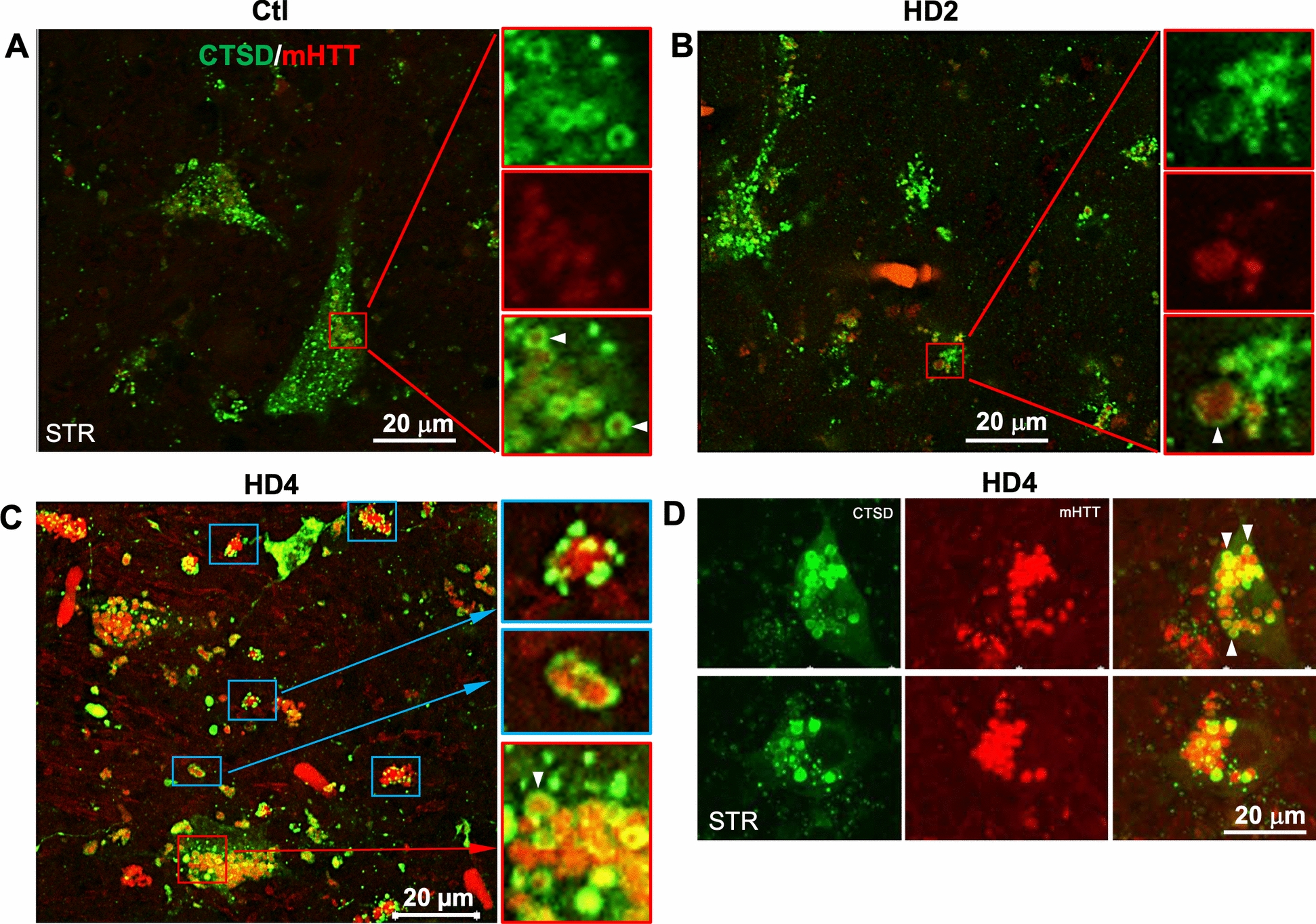
Association of mHTT with CTSD IR during disease progression. **A**–**D** Representative confocal images taken from the STR from control (A), HD2 (B) and HD4 (C, D) cases double labeled for mHTT (mEM48) and CTSD, depicting association/colocalization of mHTT IR in CTSD positive vesicles (AL/LY), where CTSD signal strongly decorates the rim of the lumen (arrowheads). CTSD signal also decorates mHTT-positive neuritic inclusions in the neuropil (blue boxes), in the forms of either discrete puncta (C, upper Inset) or a continuous ring (C, middle Inset). (D) depicts grossly enlarged (note that D has the same magnification as A-C) and clustered vesicles at HD4 stage, positive for CTSD or mHTT or both. Bars = 20 µm for (A-D)

Considering the occurrence of lysosomal pathology in HD brains appearing late in the pathology, we next evaluated possible relationships of the lysosomal abnormality with the potential substrates (e.g., HTT) reaching LYs through the autophagic process assayed using immunofluorescence. Double immunolabeling for CTSD and HTT (mEM48) in striatal neurons, the majority of which are medium spiny neurons (53, 56), from control cases revealed moderately stained CTSD puncta (Fig. 5A). HD neurons revealed mHTT immunolabeling with mEM48 that was circumscribed by CTSD (Fig. 5B, C) red boxes, and 5D, arrowheads). Also, at HD4, a splotchy CTSD staining pattern was evident by the fluorescent labeling (Fig. 5C), similar to that revealed by DAB staining shown above (Fig. 4C, E), and CTSD and/or HTT positive vesicles in many cells had become grossly enlarged and clustered (Fig. 5D). The relatively weak intraluminal HTT signal at HD2 (Fig. 5B) vs. the strong signal at HD4 (Fig. 5C, D) may imply a relatively competent lysosomal degradative function in clearing HTT in the early stage and a progressive disruption in the clearance of this substrate in overtly degenerating neurons during disease progression. At HD4, HTT-positive inclusions in the neuropil were numerous and often larger (Fig. 5C, blue boxes), colocalized with (psuedocolored yellow), or surrounded by CTSD IR puncta, suggesting that the HTT inclusions were too large to be contained within individual AL. These structures in the neuropil may correspond to the neuritic inclusion shown in Fig. 1G where AV exist inside or surrounding a large inclusion as shown in an earlier study in dystrophic neurites of the hippocampus in AD (44).

The lysosomal pathology in affected neurons in late-stage HD brains (Figs. 4, 5) involved the accumulation of CTSD positive vesicles, which were decorated by HTT (Fig. 5B–D) and concentrating in one pole of the neuron (Figs. 4C, E and 5D), a distribution pattern associated with lipofuscin granules. This was consistent with the abundance of lipofuscin granules and clusters seen under EM (see below).

## Further ultrastructural analyses of cytoplasmic vesicular organelles

The above single and double immunostaining demonstrate that a dominant vesicular pathology in affected neurons of late-stage HD that involves the accumulation and enlargement of CTSD positive vesicles which correspond to AL/LY and lipofuscin granules. We then extended our EM analysis to verify the incidence of these structures and to further assess autophagy related or unrelated vesicular structures in brain samples from a HD4 case with exceptional morphological integrity, in addition to the previously described details of cytoplasmic inclusions shown with EM in Fig. 1K.

**Fig. 6:**
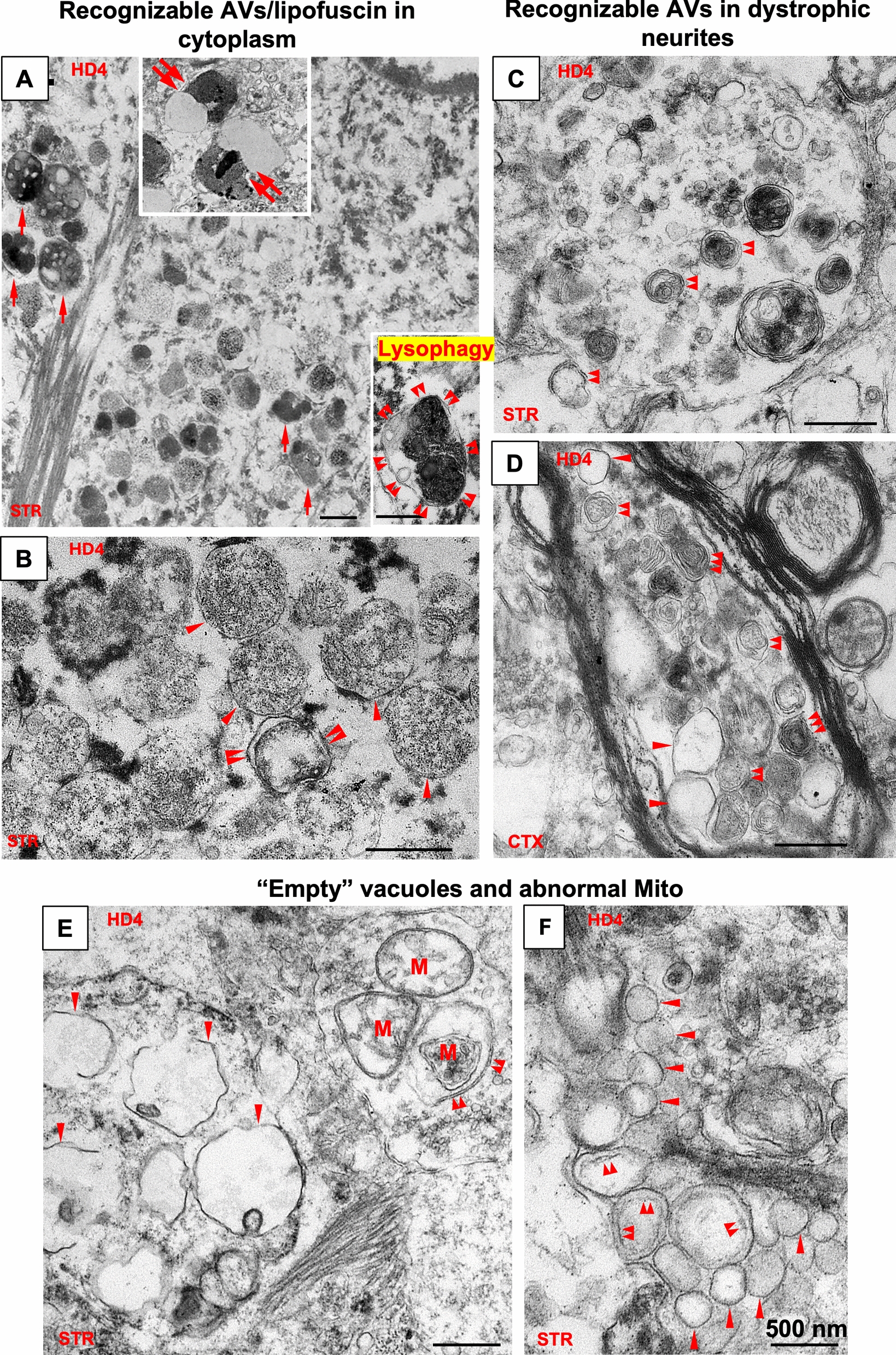
Membranous/vesicular pathologies in the later stages of HD–Accumulation of vesicles with various types of intraluminal features. Representative EM images from the STR and the CTX (as depicted on the individual panels) of an HD4 case demonstrating cytoplasmic membranous/vesicular pathologies. (**A**, **B**) Representative EM images depicting recognizable cytoplasmic vesicles of the ALP, including mature lipofuscin granules with bipartite protein/lipid morphology (A, upper Inset, double arrows), early forms of lipofuscin granules (A, single arrows), double (double arrowheads) or single (single arrowheads) membrane-limited vesicles (B), presumably corresponding to AP and AL respectively. The lower Inset in A depicts a compounded dense AL/lipofuscin granule within a double membrane sac, implying lysophagy. Bars = 500 nm. (**C**, **D**) Representative EM images of dystrophic neurites depicting recognizable AV including AP with double limiting membrane (double arrowheads) and multilamellar bodies (MLB, triple arrowheads). Clear vesicles with apparent single limiting membrane within the same dystrophic neurite (D) are indicated by single arrowheads. Bars = 500 nm. (**E**, **F**) Representative EM images for other vesicular structures of unidentified origins, including single membrane-limited clear vesicles of varying sizes with minimal intralumenal contents (E, F, single arrowheads), similar clear vesicles exhibiting apparent double membrane (F, double arrowheads. Note that the inner membrane is faint, questioning a fixation artifact, see Text). The double arrowheads in E point to a double membrane vacuole containing a mitochondrion. Bars = 500 nm

First, we verified that the cytoplasm of affected neurons contained a range of AV (AP, AL, LY) (< 500 nm) and abundant lipofuscin granules (which could also be referred to as pigmented AL)(57) with either typical bipartite lipid/protein morphology (double arrows in Fig. 6A, top inset; also see Fig. 1A, CTX, top) or clusters of more amorphous granules with heterogeneous content of varying electron density corresponding to early forms of lipofuscin granules (Fig. 6A, arrows), all reflecting incomplete degradation of lysosomal substrates within. Some of these small lipofuscin granules or granule clusters were surrounded by a double membrane suggesting attempts by the cell to digest these structures by lysophagy (58), a process of AL/LY autophagy (Fig. 6A, bottom inset). Second, of particular note were the collections of single membrane-limited 300–500 nm vesicles in some affected neurons, exhibiting a relatively uniform content of mainly granular and fibrous material (Fig. 6B, single arrowheads) resembling in part the components of large fibrous cytoplasmic inclusions described above (Fig. 1K, left). This population of vesicles, presumably AL, coexisted with double membrane-limited structures, presumably AP (Fig. 6B, double arrowheads) in the perikarya, implying no evident block in AP-LY fusion or generation of AL. The large number of these AV in some neurons supports the concept that either the generation of AP/AL has increased or more consistent with our other data, that clearance of AL has decreased. Third, in dystrophic neurites (Fig. 6C, D), which were not frequent in the HD brain, AV were clearly identified present as double-membrane bound vacuoles (i.e. AP) or multilamellar bodies (MLB) containing low electron-density intralumenal content, consistent with successful sequestration and delivery of autophagy cargos along the ALP.

Notably, there were clusters of clear vesicles of varying sizes characterized by their minimal intralumenal content (i.e. “empty”), most of which were single membrane-limited (Fig. 6E, F), single arrowheads; also see Fig. 6D), while some exhibited a double membrane appearance (Fig. 6F, double arrowheads), raising the possibility that the latter type represents the “empty AP” due to defective cargo sequestration (39). On the other hand, however, mitochondria were seen in double membrane vesicles (Fig. 6E, double arrowheads), implying that at least sequestration of this cargo, is unimpeded.

## Immunoblotting of lysosomal markers suggests upregulation of lysosomal biogenesis in the STR

To survey ALP-related biochemical changes in HD brain regions demonstrating regional changes we analyzed protein markers of the different phases of the ALP (i.e., autophagy induction signaling, AV formation and lysosomal substrate clearance) on staged brains from HD2—HD4 and control cases compared in STR and CTX using immunoblotting, again noting these are the same samples assessed by transcriptomic assays (see Table 1 Demographics). Notably, the majority of marker proteins for the early phases of the ALP, including p-p70S6 (as an indicator for mTOR inhibition), BECN1, ATG5, ATG7 and LC3, showed no changes apparent with disease progression in these regions (Fig. 4M, N; Fig. S3), in line with qPCR results shown above.

**Fig. 7:**
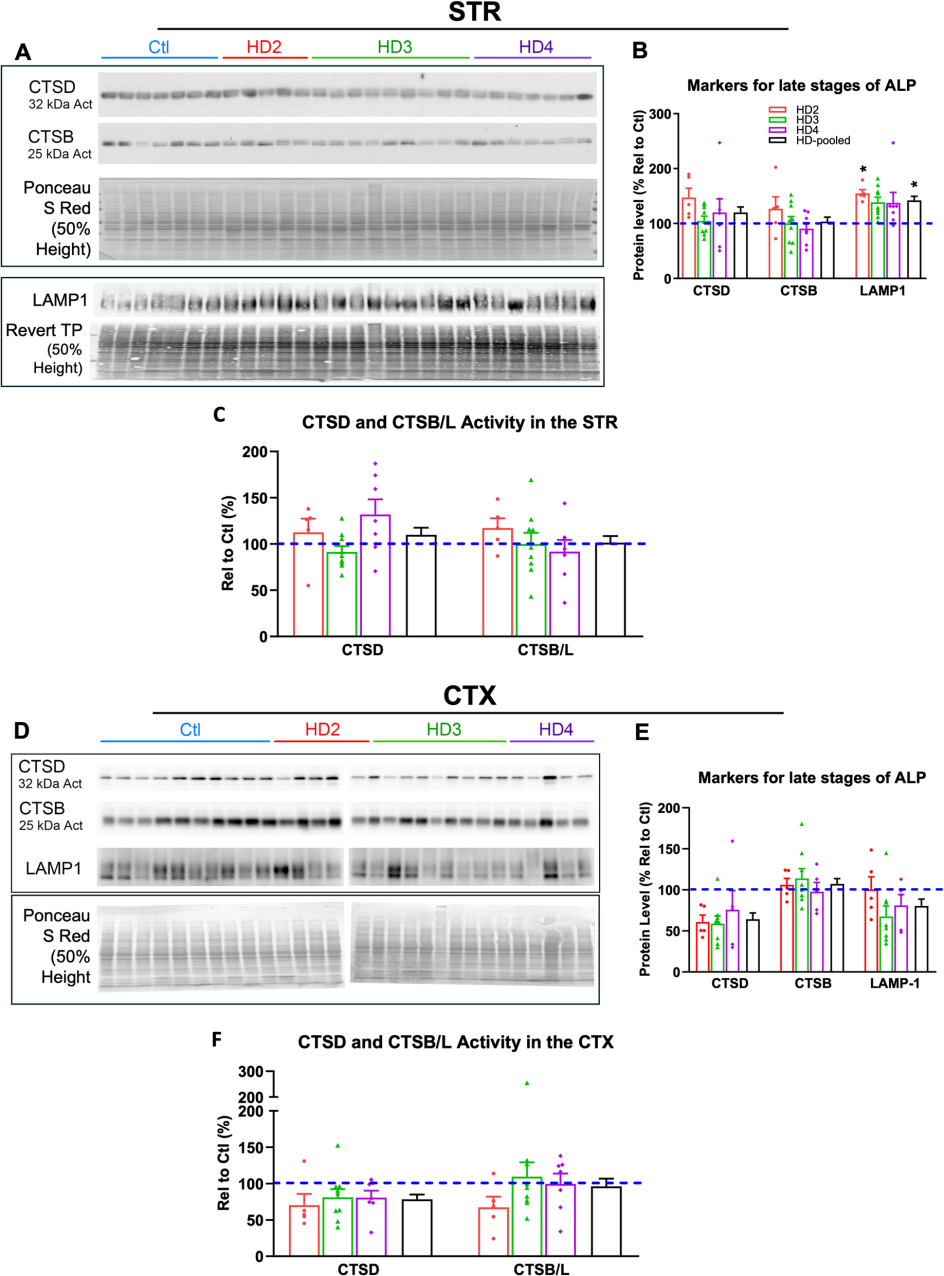
Levels of proteins involved in the late stages (e.g., lysosomal clearance phase) of the ALP and enzymatic activity assays of cathepsins in the STR and the CTX. (A and D) Representative western blots of striatal (A) or cortical (D) lysates demonstrating the levels of proteins involved in the lysosomal clearance stages of the ALP in control and HD cases. For loading, identification details and sample sizes see Fig. 3 legend. (B and E) Bar graphs for quantitation results (obtained after normalization using a specific loading control, i.e., ACTB bands, and/or bands on uncompressed original Ponceau S Red stained blots or Revert Total Protein Stain blots for each specific antigen) for the blots shown in (A) and (D), respectively. Each bar represents the result of either HD2, HD3, HD4 or the pooled data from all HD samples and is expressed as “% relative to Ctl” (set as 100% depicted by the dashed line) ± SEM. For B Ctl = 100 ± 9.808, 14.71, or 7.059 for STR CTSD, CTSB, and LAMP1. For E Ctl = 100 ± 14.71, 6.133, or 7.797 for CTX CTSD, CTSB, and LAMP1 respectively (C and F) Quantitation of CTSD and CTSB/L enzymatic activity in striatal (C) or cortical (F) lysates of controls or HD cases. Each bar represents the result of either HD2, HD3, HD4 or the pooled data from all HD samples and is expressed as “% relative to Ctl” (set as 100% depicted by the dashed line) ± SEM. Significant differences were analyzed by One-way ANOVA followed by post hoc Tukey’s multiple comparisons test. * symbols: comparisons of each bar/group with the Ctl. N of samples defined in Fig. 2

Next, for markers relating to lysosomal degradative functions at the later phase of the ALP, we did not observe significant alterations in the protein levels (Fig. 7A, B, D, E), and enzymatic activities (Fig. 7C, F), of lysosomal enzymes CTSD and CTSB involved in substrate clearance in the STR (Fig. 7A–C) and the CTX (Fig. 7D–F). However, an intriguing observation was an elevation of the levels of LAMP1 in the STR in the HD brains, appearing as early as HD2 (Fig. 7A, B), which was otherwise not observed in the CTX (Fig. 7D, E), highlighting the levels of disease severity as depicted by the CTSD IHC found in HD STR compared with a lesser degree in the CTX, while the qPCR showed limited concordance, reiterating the findings of Johnson et al. comparing transcriptomic analysis with that of proteomics in AD (59). In total, the ALP appears to be fully competent in early stages of HD progression and therefore could be a good candidate for stimulation to alleviate mHTT pathology which contrasts with AD pathology where clearance fails early on leading to exacerbation of pathology.

## Discussion

Most of the knowledge about the relationship of the ALP with HD including the roles of HTT/mHTT in autophagy has been obtained from studies using cell and animal models. However, here, we analyzed a relatively large number of human brain samples from controls and HD2-HD4 patients, which has provided human brain-derived information that can expand our knowledge about the status of ALP in HD brain, its relationship to the development of mHTT aggresome/inclusion pathology, and the relevance of animal models as surrogates to characterize HD neuropathology and pathobiology. Moreover, the findings we have described have implications as support for the potential therapeutic value of specific strategies of autophagy modulation in HD.

## The status of ALP in the HD brain

At a global level, we have demonstrated in the human brain that HTT/mHTT are substrates of ALP. Moreover, in HD brain, our collective data suggest that the ALP in affected neurons is relatively competent to maintain autophagy flux clearance capacity at an early disease stage, but that at later stages, autophagy flux declines and AL are laden with substrates including HTT that accumulate. The later stage pattern is a pathological state associated with neurodegeneration in a number of major adult-onset neurodegenerative diseases (60). A gradual bidirectional pathological relationship (a “vicious cycle”) is suggested between mHTT build-up within AL and the decline in autolysosomal clearance efficacy that is likely multifactorial (60, 61). The relative competence of autophagy flux at an early disease stage of symptomatic HD contrasts with the temporal pattern in AD, where impaired flux emerges very early in pre-symptomatic disease as a result of direct impact of disease-related genetic factors on AL/LY proteolytic efficacy (44, 47, 60, 61) and autophagy failure progresses to an unusually extreme degree as disease advances (45, 62). Preservation of autophagy early in symptomatic HD suggests the potential opportunity to intervene therapeutically at this stage to stimulate autophagy flux with less concern than applies to AD where overburdening failing AL/LY and exacerbating build-up of toxic metabolites within the pathway may be counter-productive.

At the early HD2 stage, there is limited evidence suggesting a major alteration in autophagy induction and upstream steps of autophagy, such as AP formation or AP-LY fusion. We cannot, however, exclude possible impairment in the engagement of certain autophagy cargoes by adaptor proteins which might impede sequestration and reduce ALP flux. We observed varying size clusters of double membrane limited vesicular profiles containing minimal intralumenal content (Fig. 6E, F), raising the possibility that they represent the “empty AP” described previously in mouse, cell, and patient fibroblast models of HD (39). Because these patterns were also often seen in fresh postmortem brains from individuals with AD clinical diagnoses in our other studies (Fig. S4A,B,D,E), but not seen in our previously published analyses of biopsied brain from AD patients (46), we are inclined to attribute much of this pattern to postmortem and fixation artifact, mainly resulting in a swelling and vacuolization of endoplasmic reticulum. This is supported by the common observation in the HD brain of mitochondria within AP (mitophagy) and by our further immunogold labeling by an anti-calnexin antibody in HD brain which yielded a significant decoration of these vesicular membranes (not shown). Artifactual vacuolization of lipid granules in lipofuscin may also give rise to a somewhat similar membrane pattern (Fig. S4C). Thus, our studies so far neither give significant support to the presence of empty APs nor refute the notion that they may be present in HD cells and cell models (63).

Interestingly, we detected spatial differences in the early ALP responses between the STR and the CTX. In the STR, there was a decreased correlation of both CTSD and HEXA with disease progression, while increased protein levels of LAMP1 that imply lysosomal membrane expansion suggesting a possible compensatory upregulation in lysosomal biogenesis. The absence of changes in levels of upstream ALP components (e.g., BECN1, ATG5, ATG7) supports lack of deficiency in AP formation or compartment size although a caveat is the potential for glial cell autophagy systems to mask changes in neuronal levels in immunoblot analyses at the brain tissue level.

The less vulnerable but still affected CTX displayed no significant alterations in all the ALP protein markers examined by immunoblotting and in enzymatic activities of lysosomal proteases although a number of RNA markers appeared to be elevated to a varying degree, suggesting that an apparently normal ALP machinery is maintained in this brain region. Such biochemical changes, together with the mild morphological changes [e.g. AL clustering revealed by CTSD IHC (Fig. 4, top), inclusion formation revealed by mHTT IHC with mEM48 (Fig. S1c)], and minimal HTT fragment generation (Fig. 3B), highlight the bifurcation between CTX and STR relative to the severity of pathological changes.

Neuronal autophagy occurs at both perikarya and neurites. In the latter location, AP usually form at the terminals of neurites, are transported retrogradely towards the cell body and eventually fuse with LY (and/or vesicles containing degradative lysosomal enzymes) at the proximal portion of neurites and/or the perikarya (64, 65). In line with this, we observed HTT signal within CTSD positive AL in both neuronal soma (Fig. 5B–D) and neuropil (Fig. 5C, the second inset), suggesting HTT as a cargo of autophagy. In this respect these inclusions share a strikingly similar morphology that is analogous to AD neuritic dystrophy observed by Bordi et al. (44) in AD hippocampus with vesicular structures filled with LC3 rather than HTT and circumscribed by a ring of vesicles that are filled with lytic CTSD. That these appear in the more highly vulnerable regions of striatum in HD and hippocampus in AD may prove to be a point of commonality for advanced HD with AD where clearance of substrates becomes compromised. Additionally, in neurons at late stages of disease, HTT/mHTT-containing CTSD-positive AL are abnormally enlarged and clustered. The continuing accumulation of these protein aggregates in the HD brain, especially in the STR, may be explained as a result of continuing overload of the aggregation-prone proteins on to the neurons which is beyond the degradative capacities of both macroautophagy (this study) and chaperone-mediated autophagy, which is known to play a role in HTT clearance and be upregulated in experimental HD models (66). Consistent with this, larger mHTT inclusions were also observed in the neuropil without being completely contained within AL (Fig. 5C, the first inset), suggesting that increasing amounts of mHTT accumulation at the late stage of disease progression led to the formation of aggregates outside the confines of vesicular structures that are too large to be processed by autophagy. However, we cannot exclude a scenario wherein impairments in the interactions of adaptor proteins with autophagy cargoes could lead to slower rates of clearance of substrates including mHTT (34, 35).

## The classification of inclusion bodies

We observed nuclear, neuritic and cytoplasmic inclusions and various subtypes in each category, particularly at the ultrastructural level. Although certain of these are described previously in literature (12, 13, 67–69), they have not been presented collectively or described systematically and therefore what we present here likely represents a relatively comprehensive collection of the inclusion types in the human HD brain. Among the NIIs, the main subtype is the pale-staining, spherical/ovoid fine granular and/or fibrous inclusion (Fig. 1A, left). This likely represents the most commonly reported inclusion type in brains of both human HD (12, 68, 69) and HD mouse models including R6/2, YAC128, HdhQ92 (70–72) and Q175 (our own study) (53). Among the neuritic inclusion subtypes we identified (Fig. 1D–J), the major subtype (Fig. 1G) also exhibited a similar aggregate ultrastructure to the aforementioned main NII subtype (i.e., spherical/ovoid fine granular and/or fibrous inclusion), along with additional AV and mitochondria inside and surrounding the inclusion. This subtype was also observed in other studies of human HD brain by HTT IEM (12, 13) and in HD mouse models (53, 73, 74). Again, similar aggregate ultrastructure of this type was also seen in some cytoplasmic inclusions (Fig. 1K, left). Together, this fine granular and/or fibrous structure of aggregates appears to represent the primary morphology, implying that the source of the aggregate material may be the same.

Another major ultrastructural feature of aggregates is a fiber-bundle subtype with variable shapes: rod- or comet-shaped or just parallelly arranged, which is more common in the neuritic inclusions (Fig. 1H–J), but can be occasionally seen in the nucleus (Fig. 1A, middle, arrowhead) and the cytoplasm as well (Fig. 1A, left, arrow). The fingerprint-like features of neuritic inclusions (Fig. 1H) were considered as fibrillary fascicles from abnormal mitochondria in a previous study (67). However, we interpret them as HTT-derived fibrillar bundle aggregates as revealed from our high-resolution EM images (Fig. 1H, enlarged images), and therefore include them within this fiber-bundle category.

These two ultrastructural aggregate types (compact fine granular and/or fine fibrous aggregate vs. fiber-bundle aggregate) apparently represent different morphologies, although they likely arise from and/or are primarily composed of aggregated mHTT, particularly N-terminal Exon 1 fragments, which is supported by the following considerations. First, both types were positively labeled by anti-HTT antibodies by IEM in both human and mouse brains, and both fine granular/fibrous and bundle structures can be detected even in the same single inclusion (12, 13, 74). Secondly, studies using recombinant or overexpressed mHTT N-terminal fragments have demonstrated that in addition to oligomers and protofibrils, there are two mature aggregated forms, i.e., short fibrils and more aggregated bundles similar to the two types of ultrastructural elements in the HD brain described above. In addition, these studies found that these multiple aggregation states in vitro were interconvertible (75–78). Experimental conditions, peptide sequence length, posttranslational modifications, and lipids, proteins and cellular membranes existing in an in vivo environment (79–81) can further influence aggregation and the final ultrastructural morphology of aggregates in vitro or in vivo.

## The significance of HTT fragmentation in HD

mHTT fragments are believed to be critical for the pathogenesis of HD and numerous studies have reported the presence of N-terminal and C-terminal fragments in human STR samples (82). Fragments may be generated by aberrant splicing of *HTT* or proteolytic cleavage of HTT (83). Particularly, more studies have focused on N-terminal fragments and revealed their pathological significance including their contribution to the formation of inclusions (70, 84–86). Multiple sites for cleavages by proteases like caspases and calpains and for posttranslational modifications have been identified in the N-terminus (87–92). In HTT-Knock-In mice, the majority of N-terminal fragments are most likely proteolytic products while the smallest fragment, i.e., the exon 1 protein, may be a product of incomplete splicing (93).

An early study had reported the presence of 40 kDa HTT fragments in cortical samples from juvenile HD patients (65- > 70 CAG) but not in those from controls which are the predominant species in the nuclear fraction (12), implying a primary role in NII formation. In our immunoblotting studies of the two brain regions, fragments of HTT (45–48 kDa) were not detected in the CTX but were readily detected in the STR where they were present at much higher levels in samples of HD patients than those of control cases. Together with the data from DiFiglia et al. (12), our data suggest region-specific, disease severity-dependent, and/or CAG length related generation of the fragments. These 45–48 kDa species can be considered as N-terminal fragments detected by the N-terminal antibody mEM48 but not by the Ab D7F7 which targets residues surrounding Pro1220 (not shown). Further, based on their size of 45–48 kDa, it is possible that they are generated from the cleavage at one of the calpain proteolytic sites, 437 (88), but not from other known calpain and caspase cleavages downstream (i.e., calpain at 469 and 536; caspase at 513, 552 and 586) (87, 88) to yield larger fragments. However, these fragments could also arise from sequential proteolytic cleavages of initial fragments generated by these proteases (88, 89). In addition, contribution of aspartic endopeptidases (86) and/or matrix metalloproteinase (at aa 402) (94) to the generation of these fragments may also be considered. On the other hand, such interpretations may not be accurate or necessary given that many posttranslational modifications may occur at the N-terminus (87) and that the gel migration of the HTT fragments are retarded by the expanded polyQ tract as mentioned previously (83, 95). All of these possibilities for fragment speciation make it difficult to establish the actual aa sequence size and the responsible protease(s). However, no matter how these fragments are generated, their specific increase in the HD STR (vs the controls) starting at HD2 may suggest their involvement in inclusion formation and proclivity to form inclusions rather than indicate only a deficit in their clearance by the proteolytic systems.

## Conclusions

Pharmacologically targeting ALP is currently being debated as a potential therapeutic strategy for HD, yet our understanding of the role of ALP in human HD pathogenesis is limited. In our investigation, we assessed, for the first time, the competence of the multiple steps in ALP in the highly vulnerable striatum as well as in the neocortex of the HD brain and controls at early and late stages of disease progression. Our analyses have yielded evidence that ALP function is unimpaired during the early symptomatic stages of HD. By contrast, evidence for failure of the lysosomal-related clearance steps of ALP contributing to progressive HTT build-up emerges mainly at advanced disease stages. Our findings underscore the importance of ALP as a clearance mechanism for clearing pathogenic mHTT in at-risk individuals and likely for delaying disease onset. Equally importantly for the clinical translation of these findings, the preservation of competent autophagy early in symptomatic HD supports a potential opportunity to intervene therapeutically during these early stages of HD by stimulating autophagy flux with pharmacological inducers, as proposed by others (36, 96). In this vein, administration of an mTOR inhibitor to 6-mo-old Q175 mice successfully normalized LY number, ameliorated aggresome pathology while reducing the levels of HTT-, p62- and Ub-immunoreactivity (53).

## Materials and Methods

***Brain tissue:*** Brain samples were obtained from the following brain banks: Harvard Brain Tissue Resource Center (HBTRC), Emory Center for Neurodegenerative Disease (ECND) and New York Brain Bank at Columbia (NYBBC). These banks use Vonsattel’s grading system of neuropathological severity to stage brains from individuals diagnosed clinically as having HD as Grade 0 to Grade 4 (HD0 – HD4) (5). Three brain regions were used in this study (STR = caudate nucleus of the striatum, CTX = prefrontal cortex, CBM = cerebellum) as indicated in Table 1 which provides detailed demographic information.

***Antibodies for immunohistochemistry (IHC), western blotting (WB):*** The following primary antibodies were used in this study. (1) from Cell Signaling Technology: tHTT rabbit mAb (clone D7F7, #5656, targeting residues surrounding Pro1220 of human HTT and detecting total HTTs), p70S6K pAb (#9202), p-p70S6K (T389) pAb (#9205), ULK1 pAb (#4773), p-ULK1 (S757) pAb (#6888, #14202; detecting S757 or S758 of mouse or human ULK1, respectively), ATG5 rabbit mAb (#12994), ATG7 pAb (#2631), ATG13 rabbit mAb (#13273), p-ATG13 (S355) rabbit mAb (#26839), VPS34 rabbit mAb (#81453), TRAF6 rabbit mAb (#8028), Calnexin rabbit mAb (#2679). (2) from Millipore-Sigma: ntHTT mAb (N-Terminus-specific, mEM48, #MAB5374, preferentially recognizing aggregated HTT)(13), ATG5 pAb (#ABC14), K48- or K63-specific ubiquitin mAb (#05–1307, #05–1308, respectively), βIII-tubulin mAb (#SAB4700544), β–actin mAb (#A1978). (3) from other vendors: BECN1 mAb (BD Biosciences, #612113); LC3 pAb (Novus Biologics, #NB100-2220), ATG9 (Novus Biologics, #B-110–56893); p62 mAb (BD Biosciences, #610832) or C-term-specific p62 Guinea Pig pAb (Progen Biotechnik, #C-1620); total ubiquitin pAb (Dako Agilent, #Z0458), LAMP1 or LAMP2 rat mAb (Developmental Studies Hybridoma Bank, University of Iowa, #H4A3 or #H4B4, respectively); CTSD sheep pAb (D-2–3, in-house made) (97); CTSD pAb (Scripps Laboratories, #RC245), CTSD mAb (CD1.1, in-house made) (65); CTSB pAb (Cortex Biochemicals, #CR6009RP), CTSB goat pAb (Neuromics, #GT15047).

The following secondary antibodies and reagents for immunoperoxidase labeling were purchased from Vector Laboratories (Burlingame, CA): biotinylated goat anti-rabbit or -mouse IgG/IgM, Vectastain ABC kit (PK-4000), and DAB Peroxidase Substrate Kit (SK-4100). The following secondary antibodies for immunofluorescence were purchased from Thermo Fisher Scientific (Waltham, MA): Alexa Fluor 568-conjugated goat anti-mouse IgG (A11031), Alexa Fluor 488-conjugated goat anti-rabbit IgG (A11034), and Alexa Fluor 568-conjugated goat anti-rabbit IgG (A11036).

***Immunolabeling of brain sections:*** Formalin-fixed tissue blocks of human brain (Table 1) were sectioned at 40 µm on a vibratome, or paraffin embedded and sectioned at 7 µm. Sections were deparaffinized as necessary. Antigen-retrieval was performed by boiling sections in sodium citrate buffer at 95 °C for 30 min. Sections were blocked and incubated in primary antibody O/N (up to 3 days in some cases) at 4 °C. Alexa-Fluor conjugated secondary antibodies were used for immunofluorescence and ABC detection method was used for immunoperoxidase labeling with DAB. Autofluorescence was quenched with 1% Sudan black (Sigma-Aldrich; St. Louis, MO) in 70% ethanol for 20 min. DAB labeling was inspected on a Zeiss AxioSkop II equipped with a HrM digital camera (Carl Zeiss, Germany). Immunofluoescent images were collected on a Zeiss LSM510 Metal confocal microscope.

***Ultrastructural analyses:*** For EM, vibratome sections of human brain (Table 1) were post-fixed in 1% osmium tetroxide. Following alcohol dehydration, sections were embedded in Epon (EMS, Hatfield, PA). One-micron-thick sections were stained with toluidine blue for light microscopic examination and ultrathin sections prepared and stained with uranyl acetate and lead citrate. Material was viewed with a Philips CM 10 electron microscope equipped with a digital camera (Hamamatsu, model C4742-95) aided by AMT Image Capture Engine software (version 5.42.443a).

Post-embedding IEM with gold-conjugated secondary antibody was performed to detect mHTT (antibody mEM48), pan-Ub, p62 and CTSD signal in neuronal cell bodies and the neuropil using a previously described protocol (88). Ultrathin sections were placed on nickel grids, air-dried, and etched briefly with 1% sodium metaperiodate in PBS followed by washing in filtered double-distilled water and incubated with 1% BSA for 2 h. Sections then were incubated overnight in the Rabbit anti-CTSD antibody (RU2, 1:1000) in a humidified chamber overnight at 4 °C, washed in PBS, and incubated in an anti-Rabbit IgG secondary antibody conjugated with 10-nm gold particles (Amersham, Buckinghamshire, UK) for 2 h at room temperature. Grids were washed and briefly stained with uranyl acetate and lead citrate before examination.

Please note that the majority of EM images were from one HD4 case which had shorter PMI and exhibited an exceptionally high level of preservation of ultrastructure compared to over 20 postmortem HD brains surveyed. We did examine additional HD4 and HD3 cases (Table 1) to survey similar info to what we found from the above HD4 case, however, their ultrastructure was suboptimal for performing quantitative EM analyses.

***Preparation of tissue extracts:*** Grey matter (0.5 g) was dissected from the STR and the CTX (Brodmann’s area 9/10) of human brains (Table 1) and homogenized in RIPA buffer (50 mM Tris/HCl pH 7.4; 0.15 M NaCl; 5 mM EDTA; 1 mM EGTA; 0.5% Sodium deoxycholate; 1% NP40, 0.1% SDS with protease inhibitors 1 mM AEBSF (Gold Biotechnology, St. Louis, MO) and 20 µg/ml of leupeptin and pepstatin (US Biochemicals, Cleveland, OH), and phosphatase inhibitor microcystin LR (1 ng/ml, Enzo). Lysates were frozen and thawed 3 times followed by centrifugation at 10,000 g for 30 min to yield a total tissue lysate supernatant. Protein content was determined by the BCA method (98). Brain lysates were examined by western blotting for various marker proteins for the ALP.

***SDS-PAGE and western blotting:*** Lysate extracts (10–40 µg total protein) were separated on 4–20% or 10% Tris–glycine SDS-PAGE gels and transferred to nitrocellulose (Pall, Pensacola, FL) for probing with antisera as noted along with appropriate external controls. Blots were blocked for 1 h at 37 °C in 1 × TBST containing 5% blotting grade dry milk (W/V), incubated in 1° Ab in block solution O/N at 4 °C, washed 3 × 10 min in TBST at RT followed by incubation with 2° Ab conjugated to horseradish peroxidase diluted in block solution for 90 min at RT. Blots were washed 3 × 10 min and immunoreactive bands were visualized with ECL reagent (RPN2209, Millipore Sigma) and the bands were quantified using MultiGauge V. 3.0 (Fuji Film) software. Target proteins were normalized following stripping and reprobing against ACTB as a housekeeping protein, and/or with bands on uncompressed original total protein-stained blots with Ponceau S Red stain or Revert 700 Total Protein Staining (Li-Cor, Lincoln, NE) prior to immunostaining.

It is important to note that lysates for regional analyses were run together, transferred to the same membrane for immunoreaction and imaging together to normalize results (see Source Data files). Exposures were approximated for multiple conditions based on levels found for external controls as noted in Results. In some cases, especially in cases of assaying housekeeping or other highly expressed antigens that exposure times are difficult to control and maintain signal linearity on film, blots were developed using DAB enhanced with nickel. Blots were stripped in 0.2 M HCl, pH 2.2 containing 0.1% SDS and 1% Triton X-100 for 1.5 h at 25 °C and reprobed using the above protocol.

***RNA preparation:*** For routine qPCR analyses 100 mg of grey matter from the STR and the CTX (Brodmann’s area 9/10) of human brains (Table 1) was dissected and extracted in 1.5 ml Trizol reagent (ThermoFisher/Life Technologies, 15,596,026) using a hand-held homogenizer (6 × 15 s bursts)(Pro-Scientific, Oxford, CT) followed by mixing with 300 µL of chloroform. Samples were centrifuged at 12,000 g for 15 min at 4 °C. The aqueous phase was collected and 750 µL isopropanol was added; samples were spun again at 12,000 g for 10 min at 4 °C. Supernatant was removed and pellet washed 2 times with 75% ice-cold ethanol and centrifuged 7500 g for 5 min at 4 °C. The pellet was redissolved in 100 µL of RNAase-free distilled water. Total RNA quality (RNA Integrity Number, RIN) was assessed on an Agilent Bioanalyzer using an RNA Nanochip (2100; Agilent Technologies; Santa Clara, CA). RNA quantity was interpolated from the Agilent chip by using an RNA ladder with a known concentration of 150 ng.

***Preparation of cDNA and qPCR:*** Starting concentrations of total RNA were normalized for samples whose concentrations were estimated on different Agilent chips. cDNA was prepared using TaqMan Reverse Transciption Reagent kit (Applied Biosystems; Branchburg, NJ) according to manufacturer’s instructions. Following reverse transcription, sample cDNA was loaded in triplicate into wells of a 96-well optical reaction plate containing appropriate target gene primer (Applied Biosystems, Branchburg, NJ). GAPDH (glyceraldehyde 3-phosphate dehydrogenase), ACTB (actin β), and HPRT1 (hypoxanthine phosphoribosyltransferase 1) were run as housekeeping genes, also in triplicate, for each sample and on the same plate, as endogenous controls. Total reaction volume per well was 20µL. qPCR was performed in the ABI Prism 7900HT Sequence Detection System (Applied Biosystems Branchburg, NJ) as described previously (99).

***Calculation of qPCR results:*** Following qPCR, the target genes were normalized against housekeeping genes GAPDH and ACTB. Results were calculated using the ΔΔCt method (Applied Biosystems, Branchburg, NJ Bulletin #2). Control values were averaged, and sample values were recalculated and expressed as percent of control. Outliers were recognized as values falling beyond two standard deviations of mean and were discarded from the analyses.

***Enzymatic assays in brain homogenates:*** CTSB and CTSL were assayed by measuring the release of 7-amino-4-methylcoumarin (amc) from Z-Phe-Arg-amc at pH 5.5 (substrate recognized by both enzymes (Enzo, Plymouth Reading, PA) modified from the method of Barrett and Kirschke (100) to utilize microplate procedures. Typically, assays were performed in black microplates in a volume of 100 µl mixture (1–5 µl of enzyme in 50 mM NA-Acetate, pH 5.5 containing 1 mM EDTA and 10 µM Z-Phe-Arg-amc). Fluorescence of amc released was read at different time points in a Wallac Victor-2 spectrofluorimetric plate reader with a filter set optimized for detection of amc standard solution with excitation at 365 nm and emission at 440 nm. The reaction was linear up to 2 h. Enzyme activity was expressed as the amount of amc released per hour per mg protein.

CTSD was assayed at 37 °C at pH 4.0 by measuring the release of amc containing peptide, 7-methoxycoumarin-4-acetyl-Gly-Lys-Pro-Ile-Leu-Phe from 7-methoxycoumarin-4-acetyl-Gly- Lys-Pro-Ile-Leu-Phe-Phe-Arg-Leu-Lys (Dnp)-D-Arg-NH2 (BioMol-Enzo, Plymouth Reading, PA), according to the method of Yasuda et al. (101). Assays were performed in black microplates in a total volume of 100 µl (0.1 M sodium acetate buffer pH 4.0 containing 20 µM substrate with and without 3 µg of pepstatin) for one hour. Fluorescence released was read in a Wallac Victor-2 Spectroflurimetric plate reader with a filter optimized for detection of amc standard solution with excitation at 365 nm and emission at 440 nm. However instead of using amc standard, a quenched standard 7-methoxycoumarin-4-acetyl-Pro-Leu-OH was used for expressing enzyme activity to account for the release of peptide containing amc instead of free amc. Enzyme activity was expressed as the relative amount of quenched standard released per hour per mg protein. The specific activity of cathepsins were calculated by calculating the ratio of enzyme activity to the densitometric data obtained from western blots for each enzyme.

## Supplementary Information

Additional file 1.

Additional file 2.

Additional file 3.

Additional file 4.

Additional file 5.

Additional file 6.

Additional file 7.

Additional file 8.

Additional file 9.

Additional file 10.

Additional file 11.

Additional file 12.

Additional file 13.

Additional file 14.

Additional file 15.

Additional file 16.

## Data Availability

All data generated or analyzed during this study are included in this published article [and its supplementary figure and Source Data files].

## Abbreviations

AD: Alzheimer’s disease
AL: Autolysosomes
ALP: Autophagy-lysosomal pathway
Amc: 7-amino-4-methylcoumarin
AP: Autophagosomes
ATG: Autophagy-related protein
AV: Autophagic vacuoles
Ctl: Control;
BECN1: Beclin-1
CTSB: Cathepsin B
CTSD: Cathepsin D
CTX: The prefrontal cortex
HD: Huntington’s disease
HTT: Huntingtin protein
IEM: Immuno-gold electron microscopy
IR: Immunoreactivity
LAMP1: Lysosomal-associated membrane protein 1
LC3: Microtubule-associated protein 1 light chain 3
LY: Lysosomes
mTOR: Mechanistic target of rapamycin kinase
mHTT: Mutant huntingtin protein
NII: Neuronal intranuclear inclusions
STR: Caudate nucleus of the striatum
p62/SQSTM1: Sequestosome 1
Ub: Ubiquitin
ULK1: Unc-51 like autophagy activating kinase 1
UPS: Ubiquitin-proteasome system
IHC: Immunohistochemistry
WB: Western blotting
