# Supplementary figures and images for: Pathobiology of the autophagy-lysosomal pathway in the Huntington’s disease brain
Source: Acta Neuropathol Commun. 2025 Nov 7;13:228. doi: 10.1186/s40478-025-02131-8 (PMC12598841; doi:10.1186/s40478-025-02131-8)

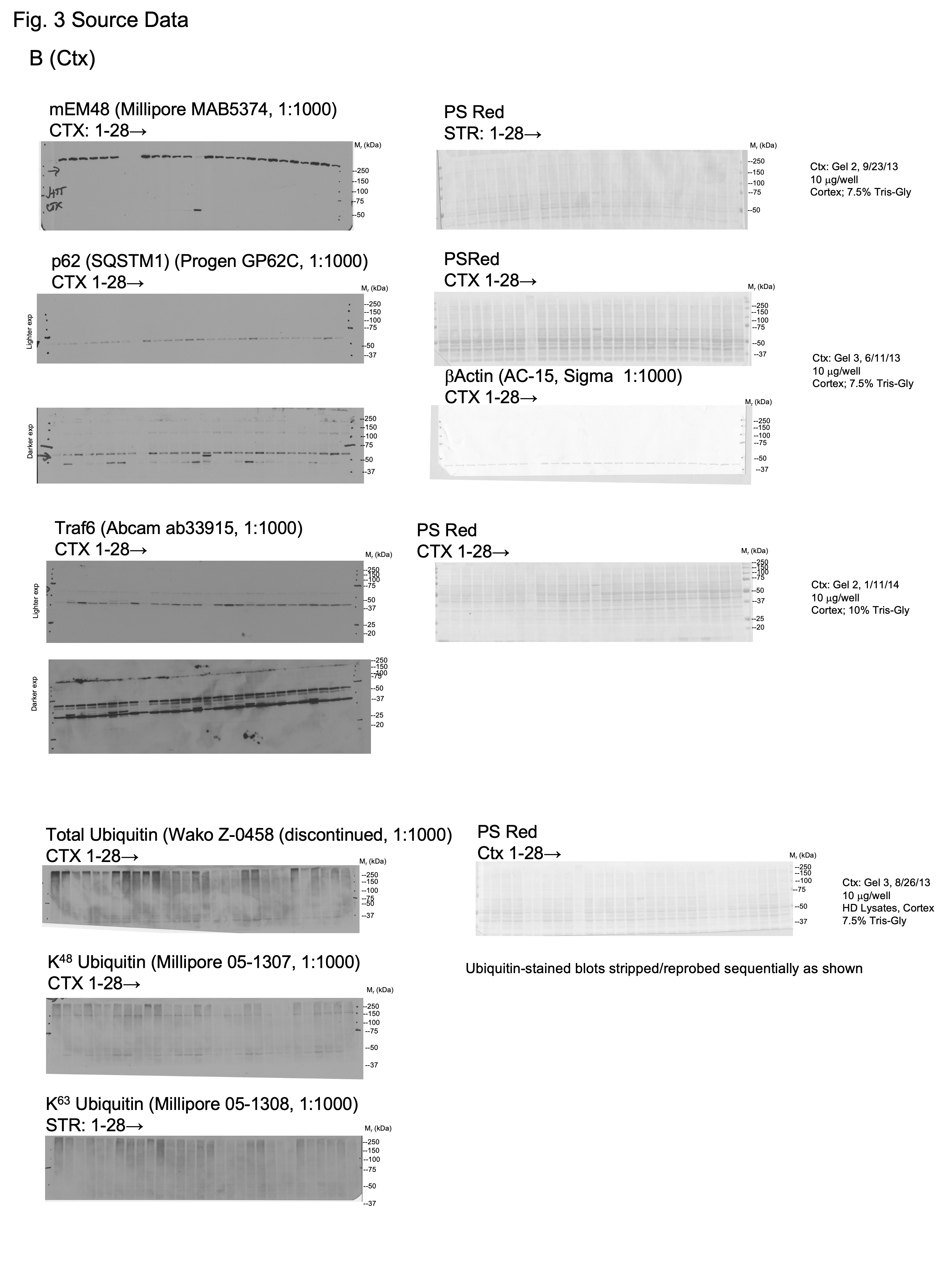

Supplement: Supplementary file 1 — Additional file 1. [file 40478_2025_2131_MOESM1_ESM.tiff]

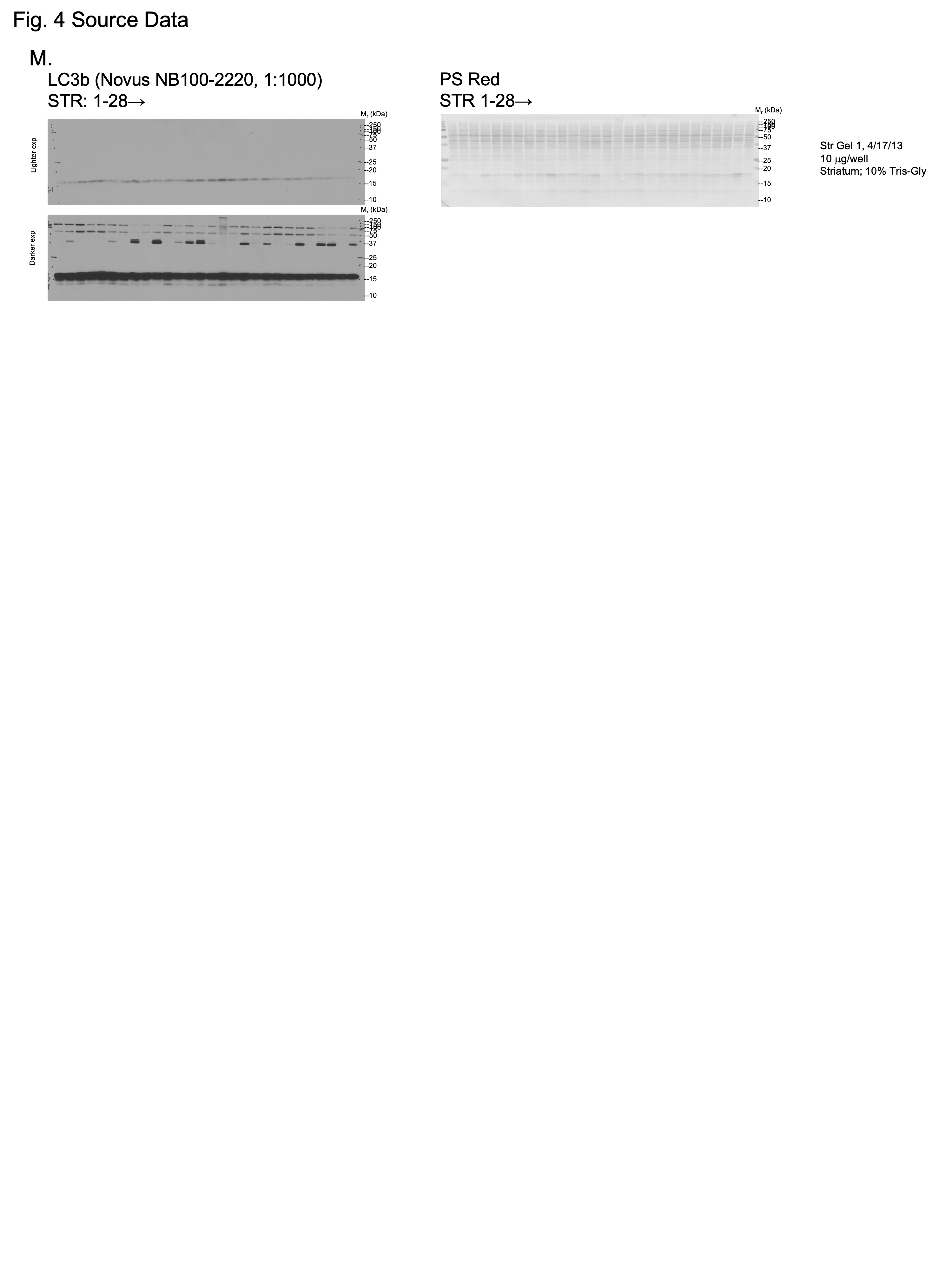

Supplement: Supplementary file 2 — Additional file 2. [file 40478_2025_2131_MOESM2_ESM.tiff]

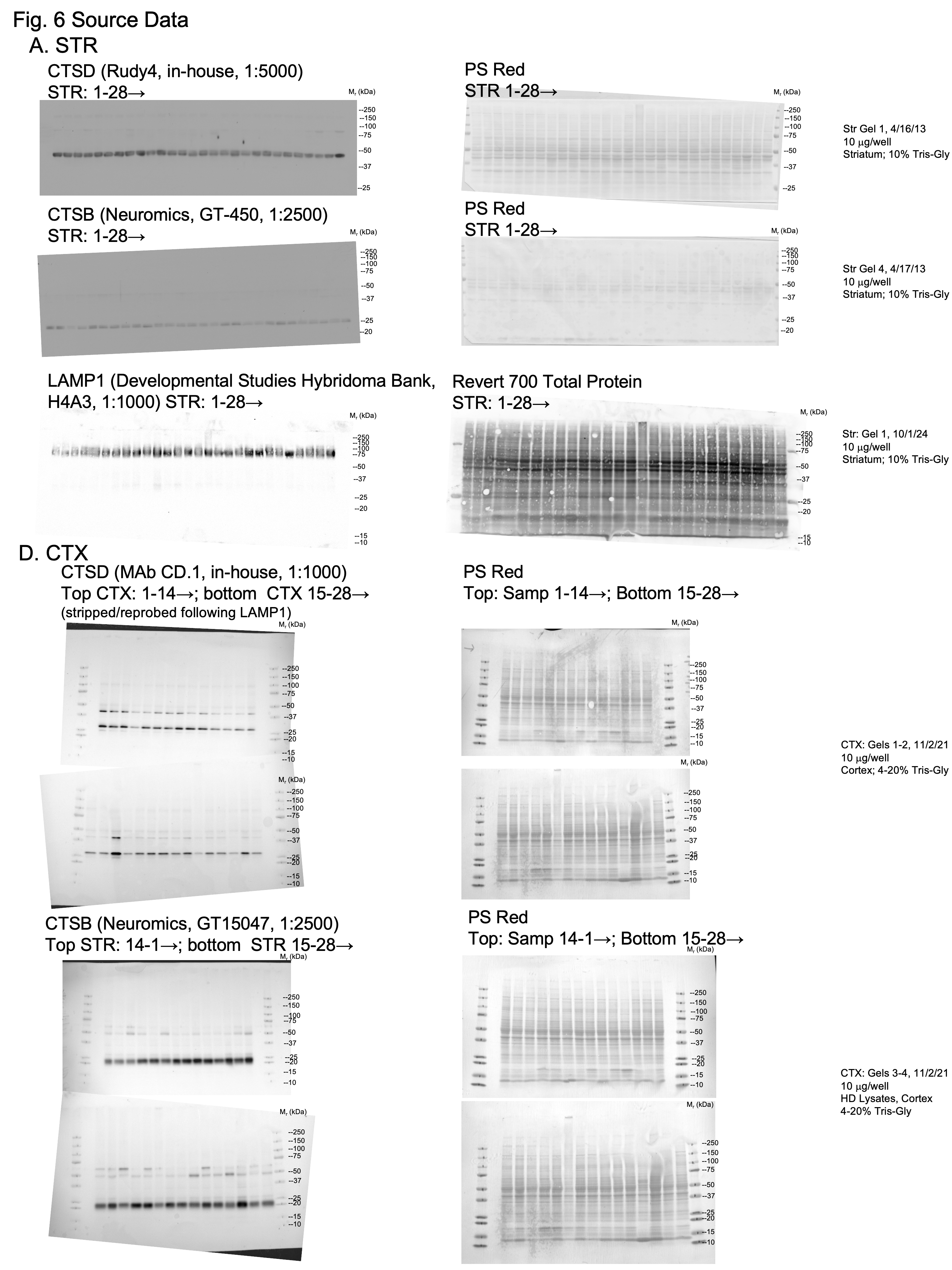

Supplement: Supplementary file 3 — Additional file 3. [file 40478_2025_2131_MOESM3_ESM.tiff]

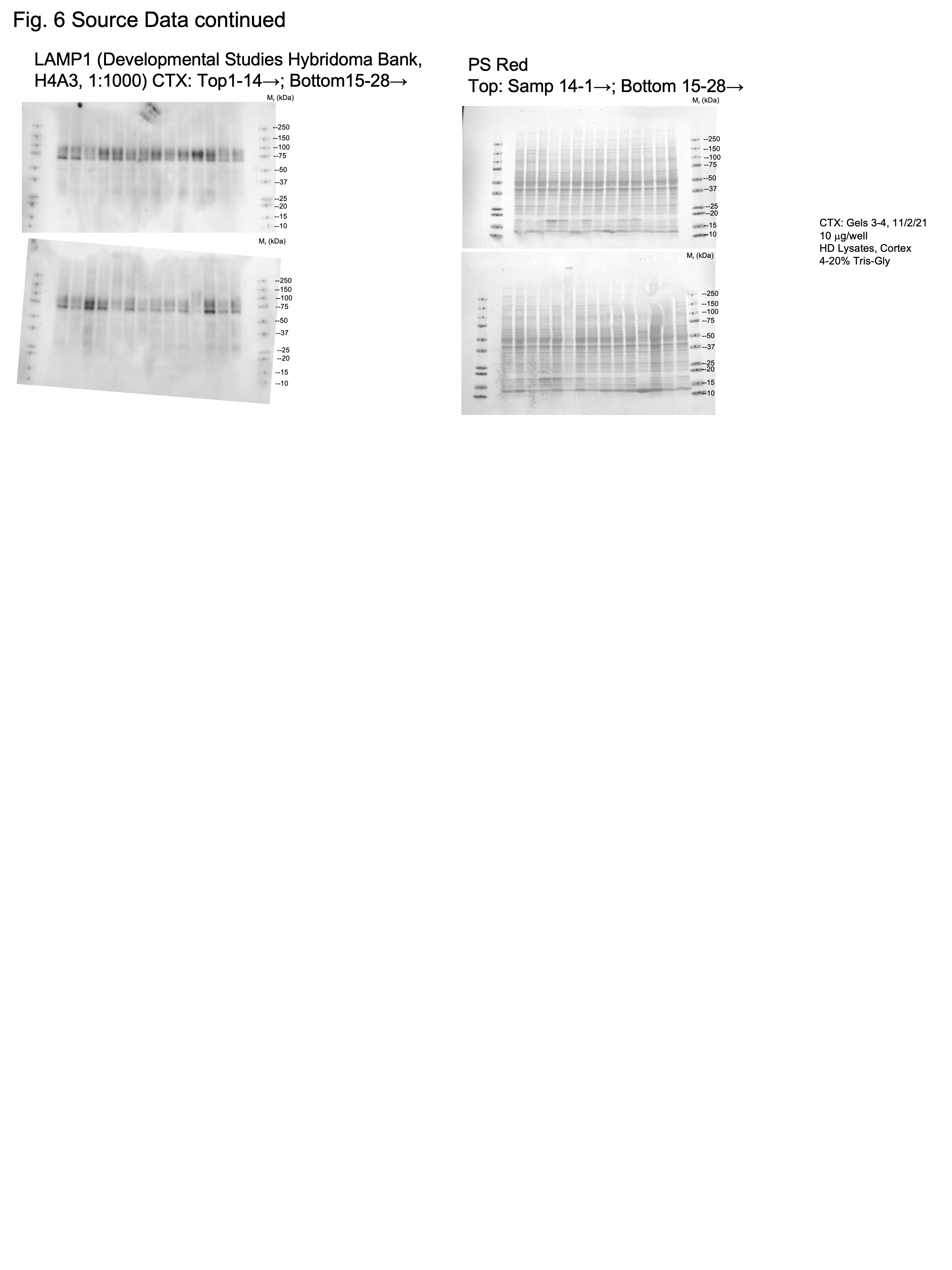

Supplement: Supplementary file 4 — Additional file 4. [file 40478_2025_2131_MOESM4_ESM.tiff]

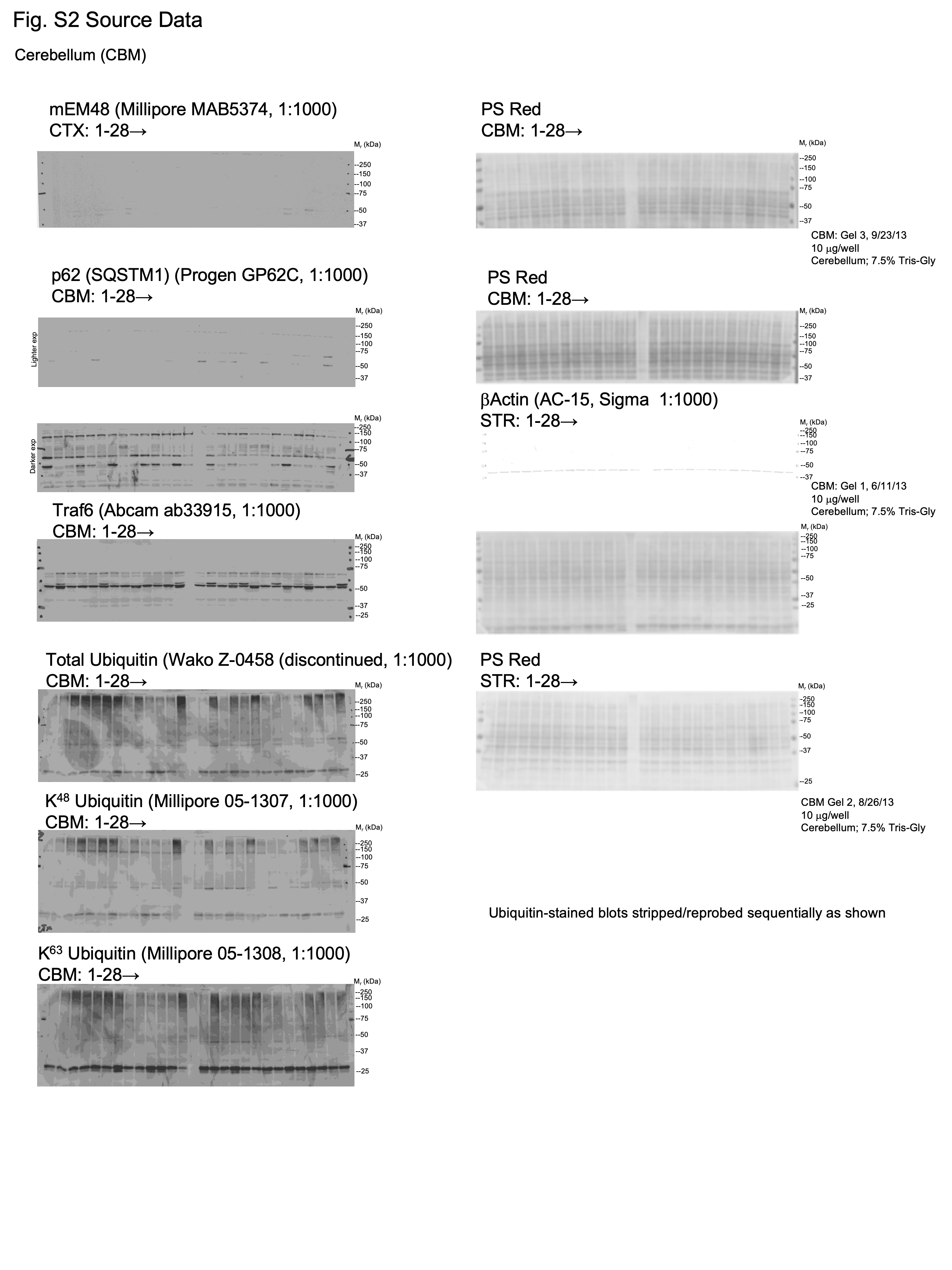

Supplement: Supplementary file 5 — Additional file 5. [file 40478_2025_2131_MOESM5_ESM.tif]

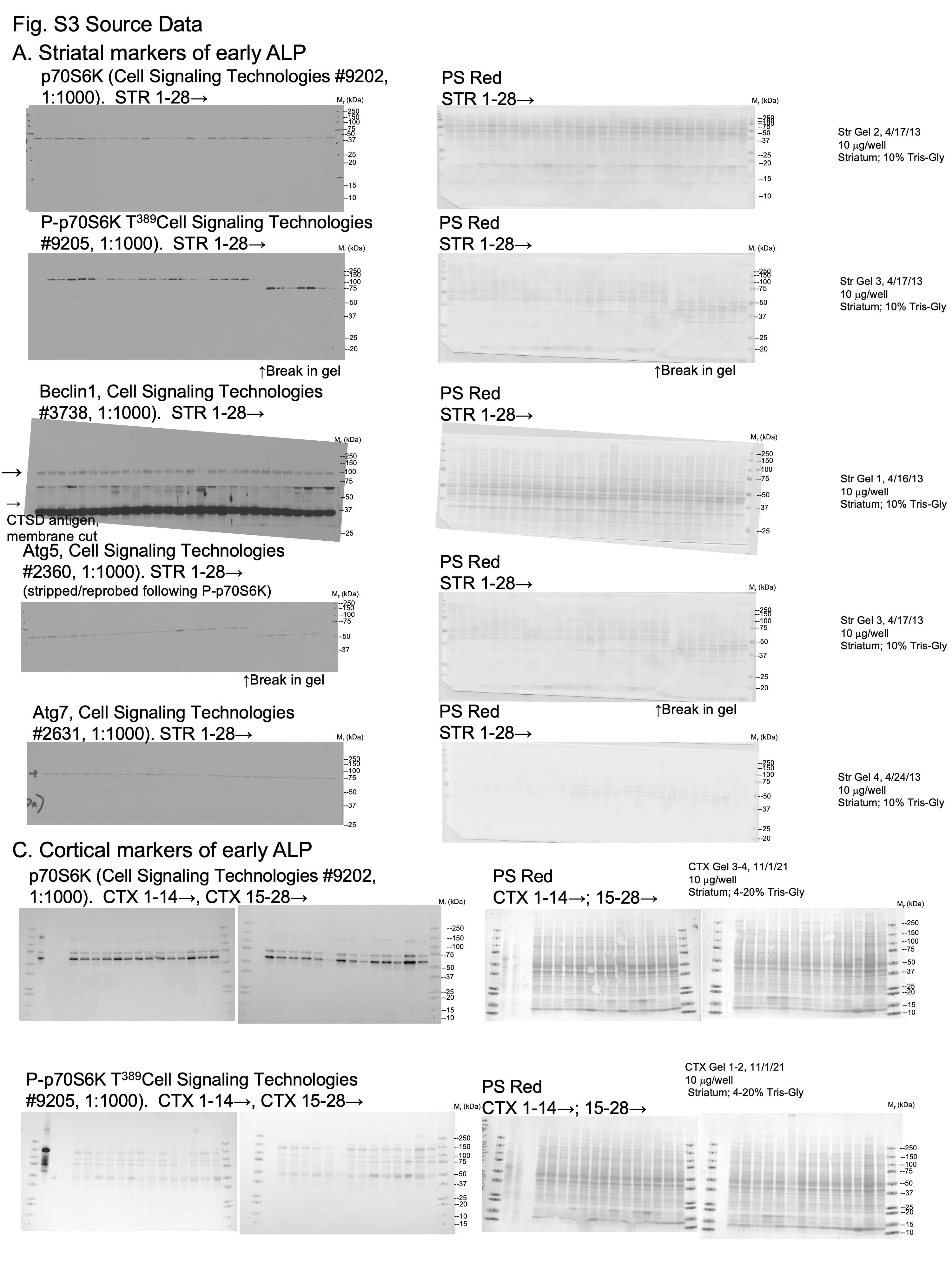

Supplement: Supplementary file 6 — Additional file 6. [file 40478_2025_2131_MOESM6_ESM.tiff]

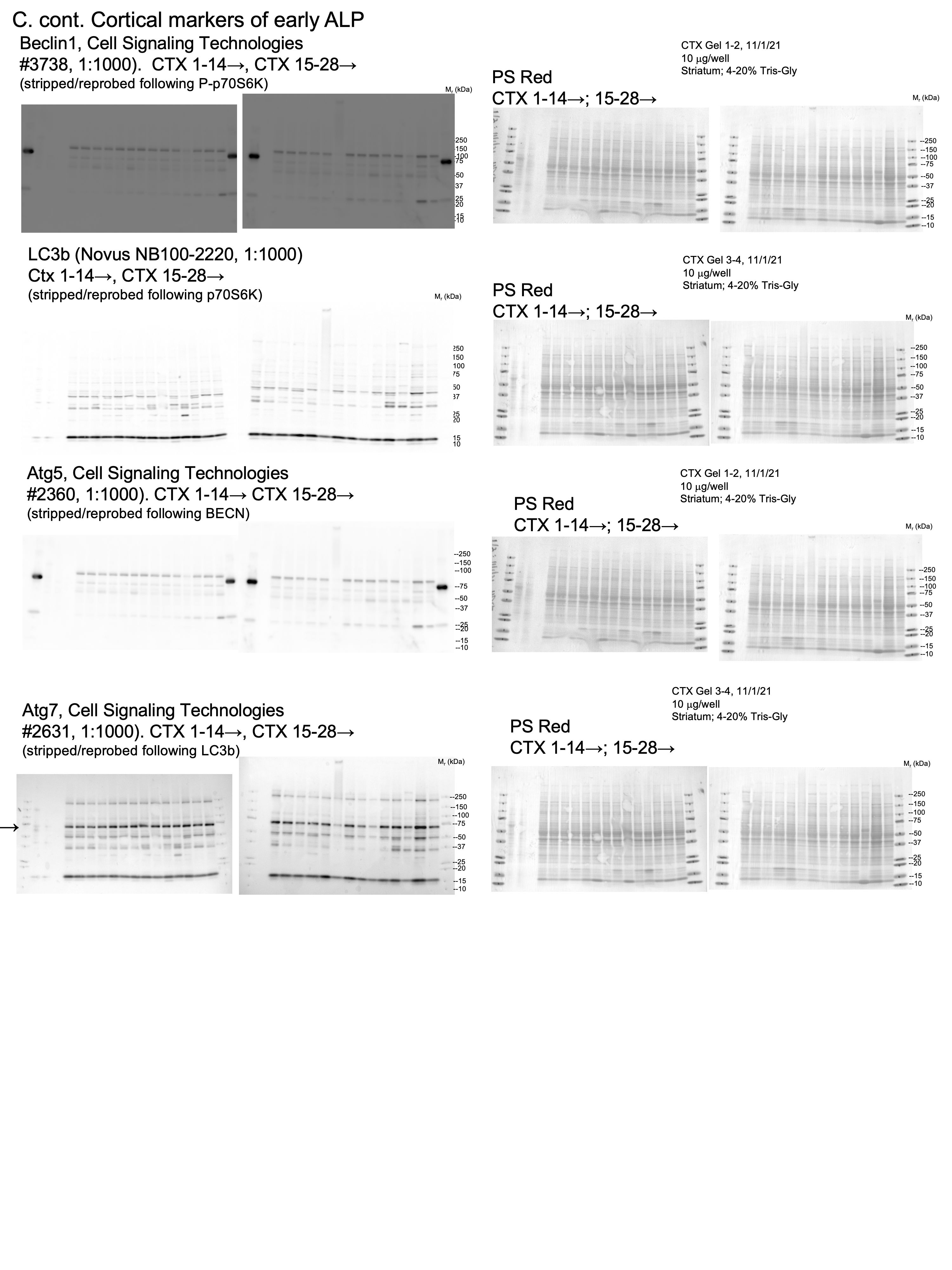

Supplement: Supplementary file 7 — Additional file 7. [file 40478_2025_2131_MOESM7_ESM.tiff]

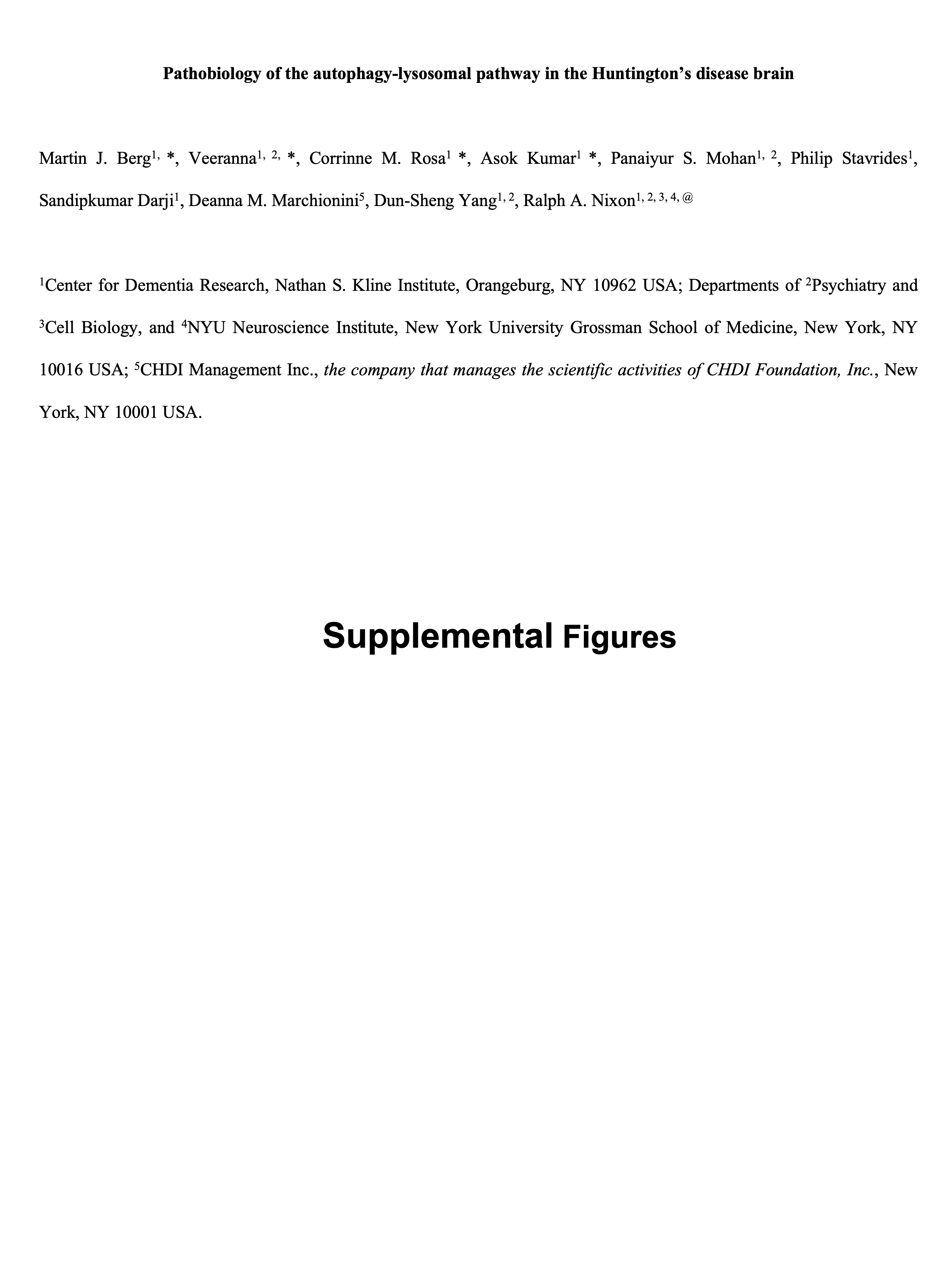

Supplement: Supplementary file 8 — Additional file 8. [file 40478_2025_2131_MOESM8_ESM.tiff]

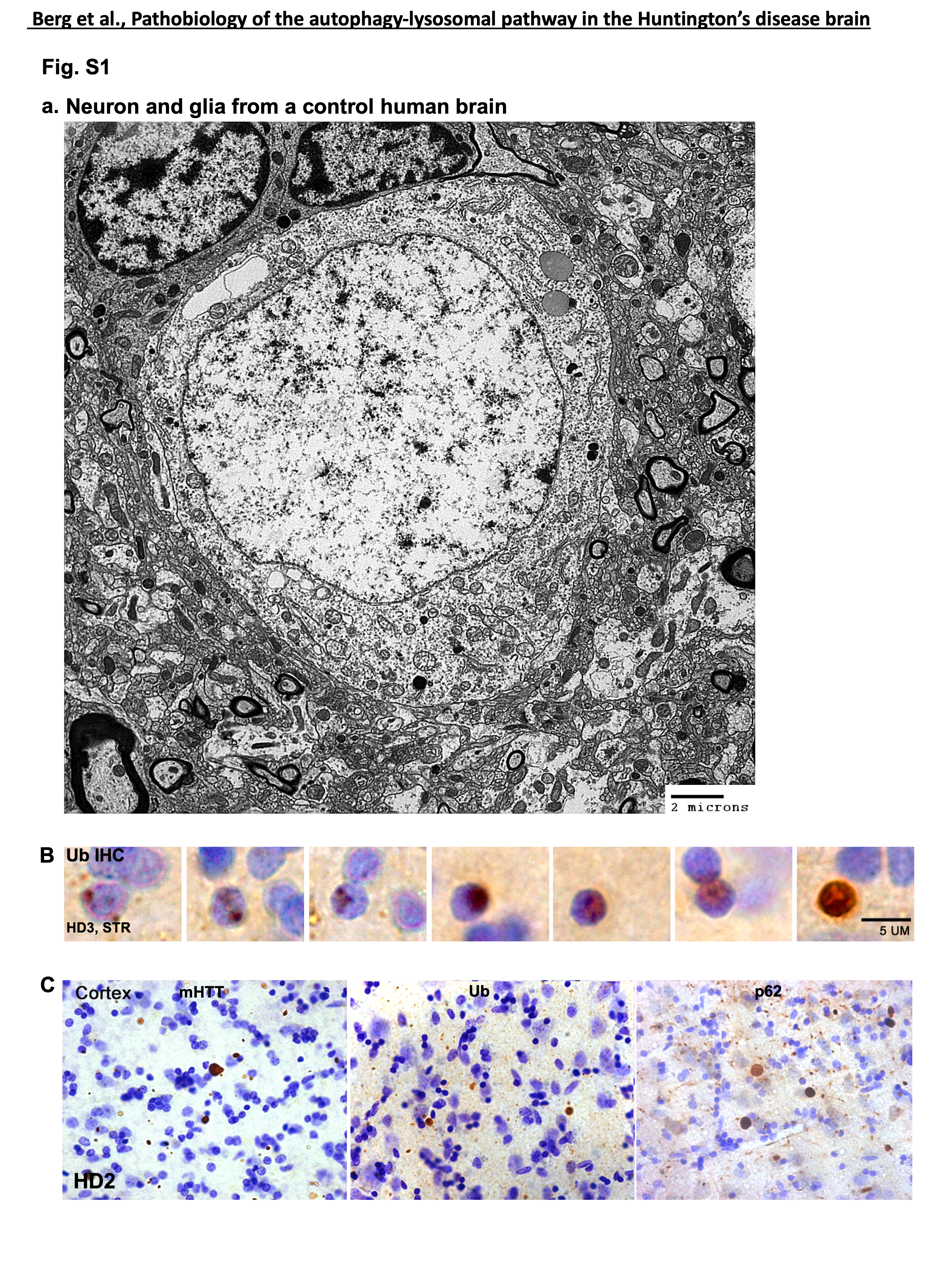

Supplement: Supplementary file 9 — Additional file 9. [file 40478_2025_2131_MOESM9_ESM.tiff]

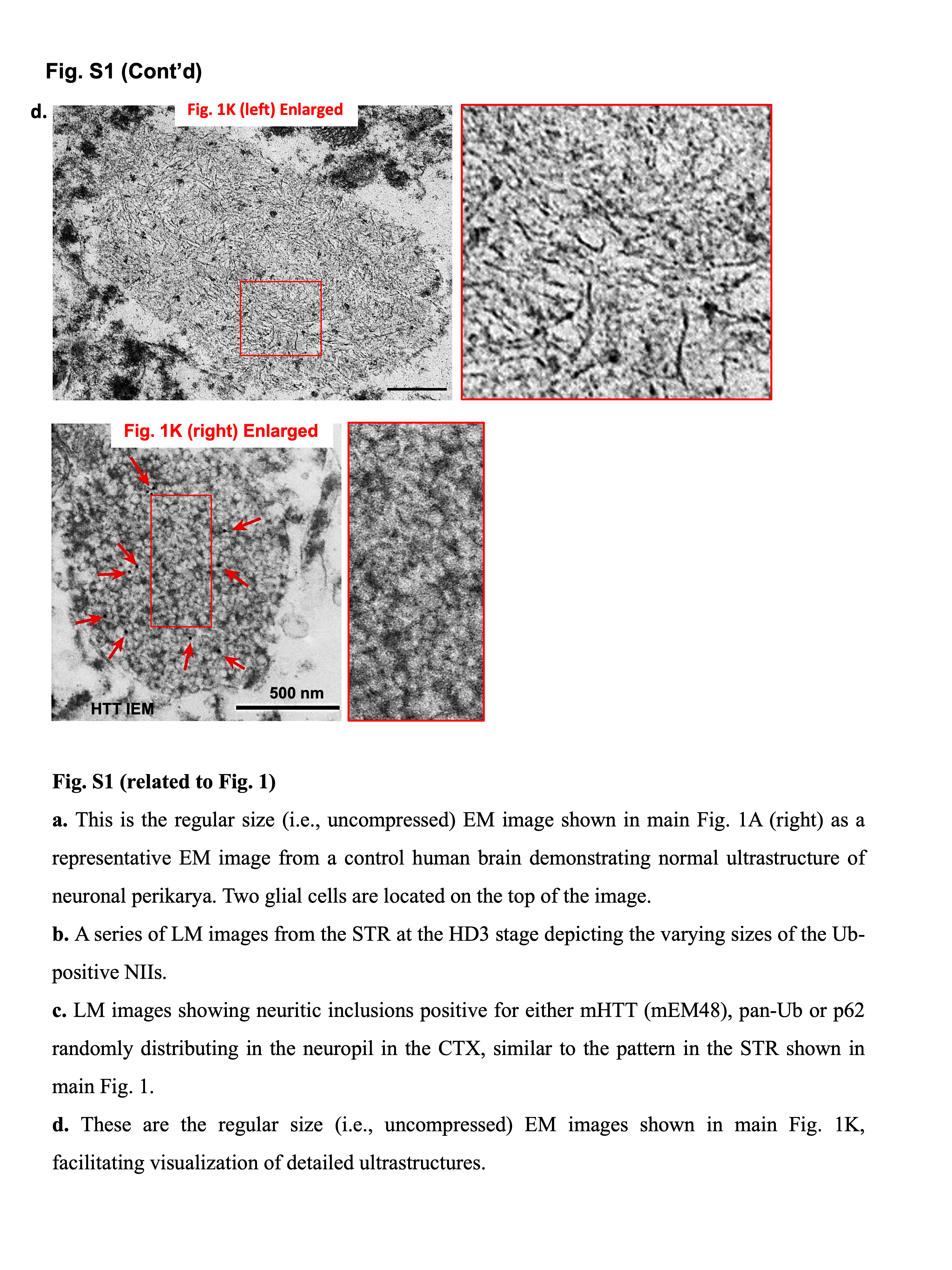

Supplement: Supplementary file 10 — Additional file 10. [file 40478_2025_2131_MOESM10_ESM.tiff]

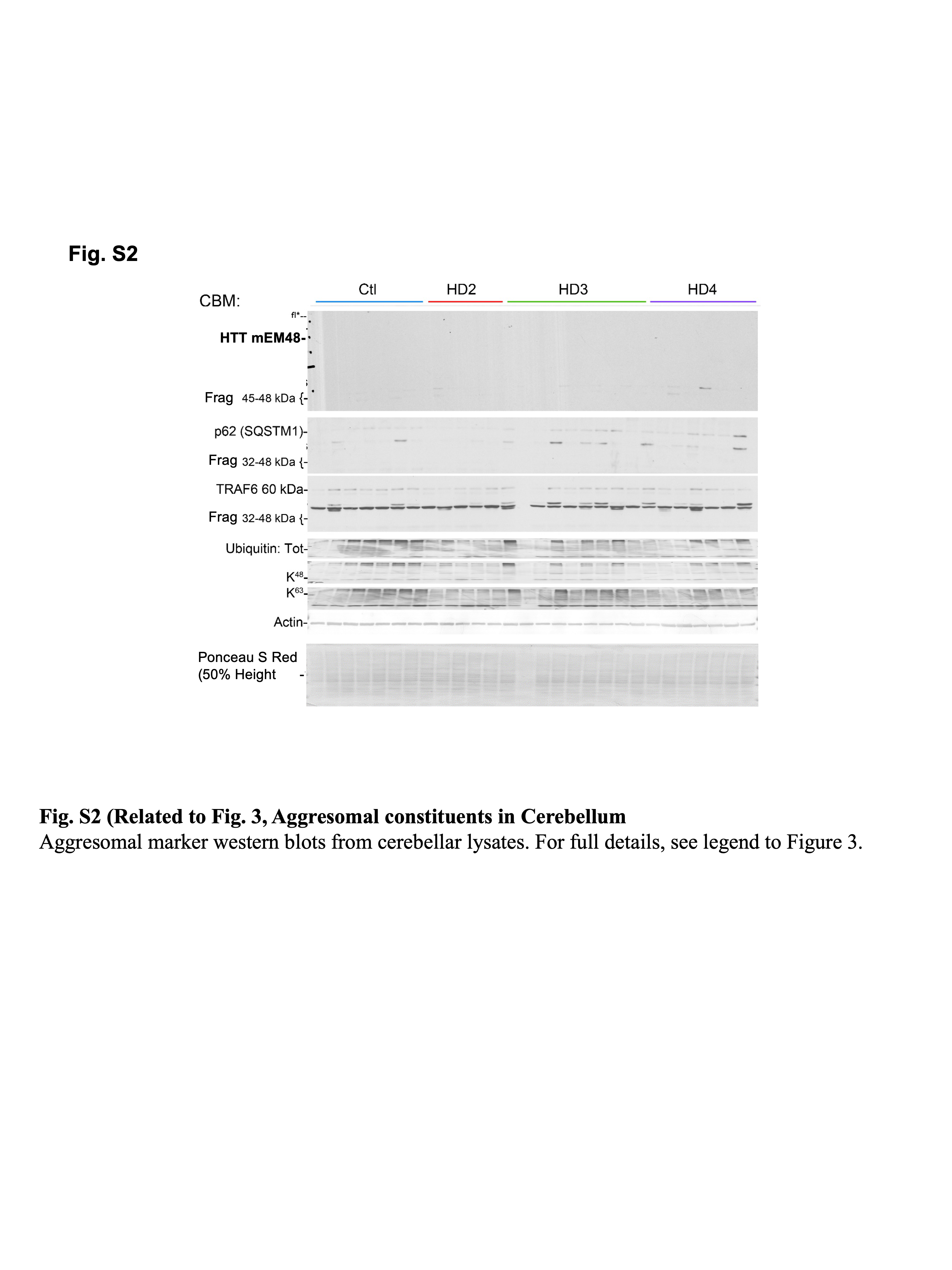

Supplement: Supplementary file 11 — Additional file 11. [file 40478_2025_2131_MOESM11_ESM.tiff]

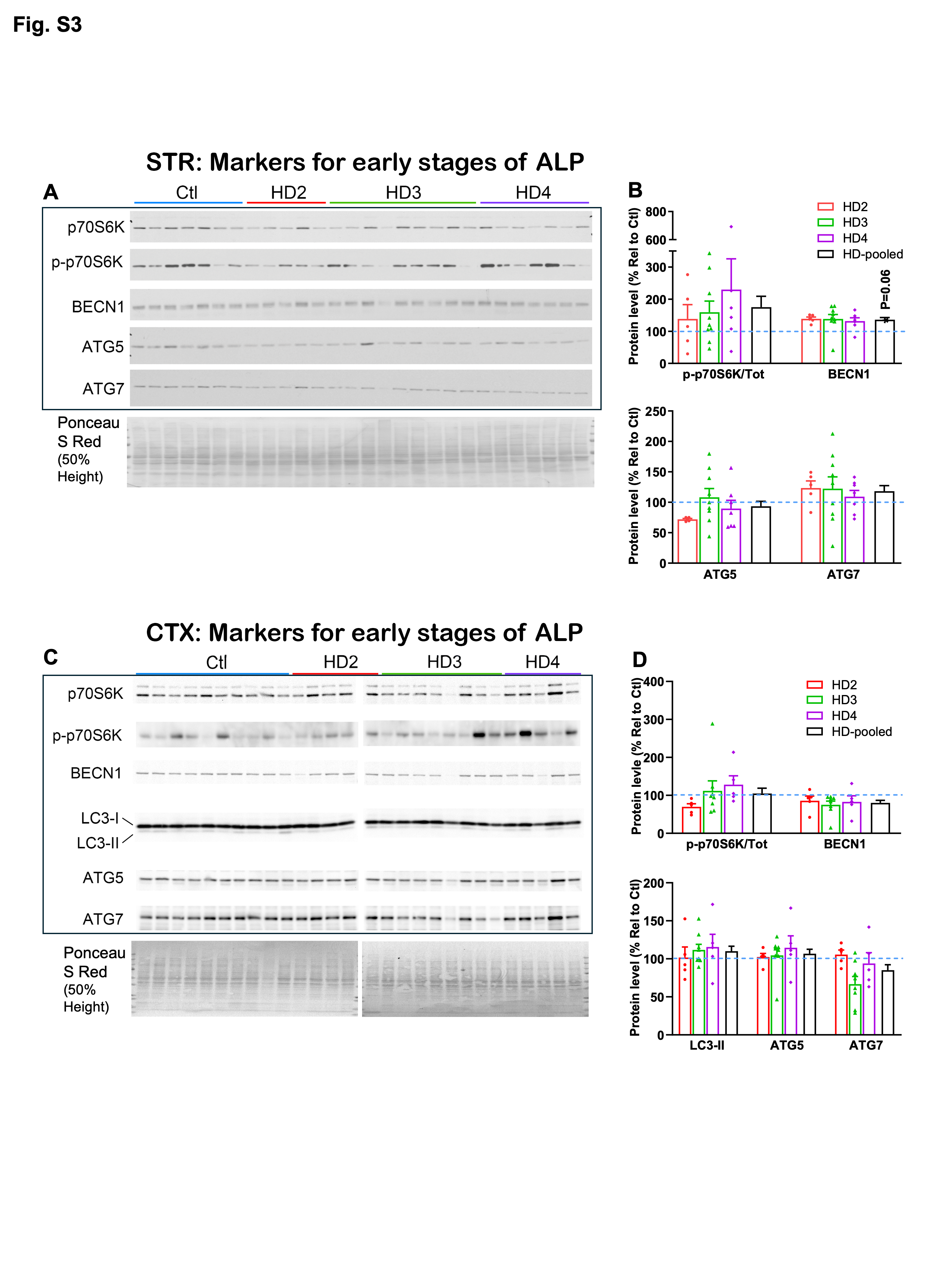

Supplement: Supplementary file 12 — Additional file 12. [file 40478_2025_2131_MOESM12_ESM.tiff]

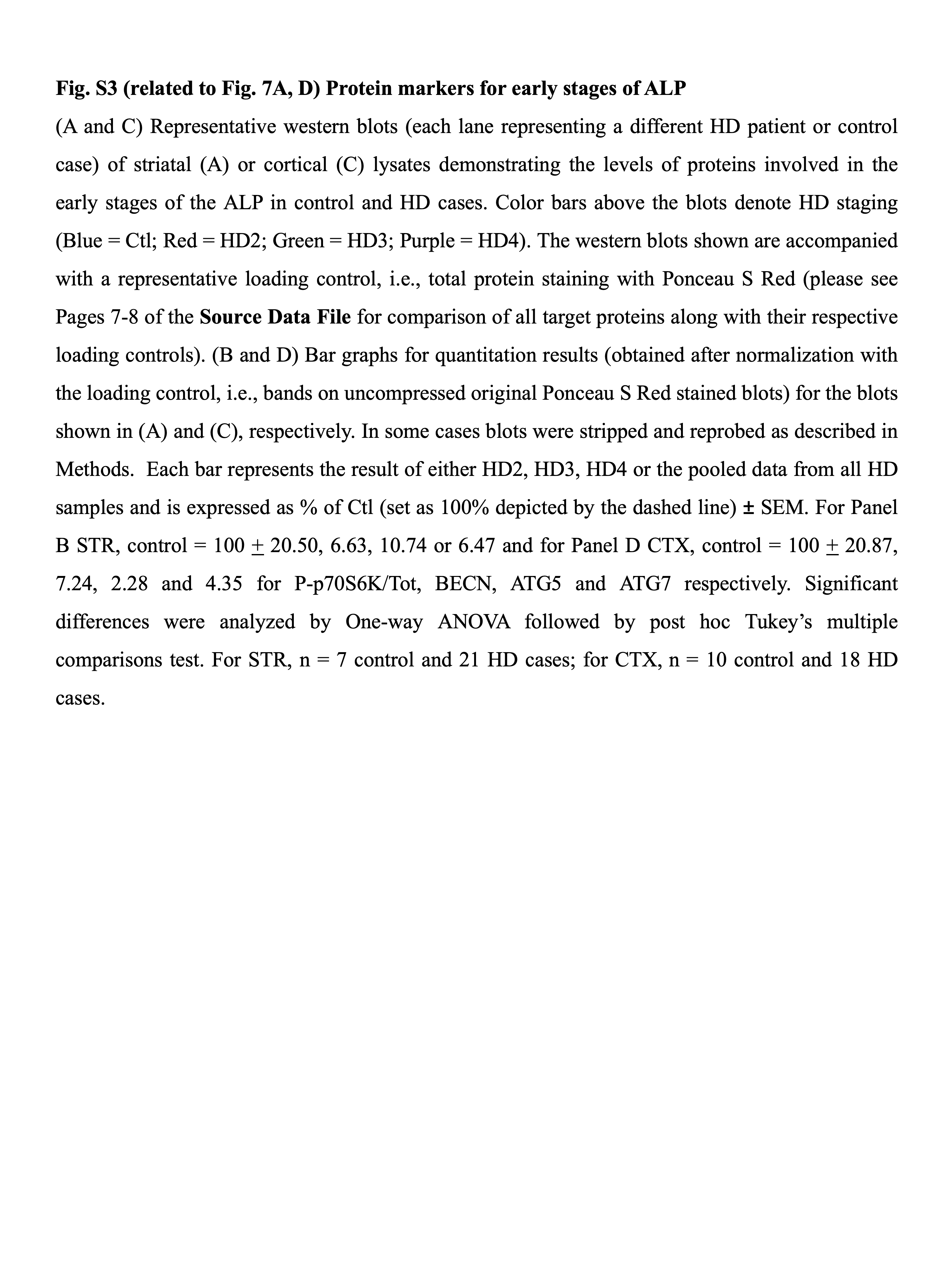

Supplement: Supplementary file 13 — Additional file 13. [file 40478_2025_2131_MOESM13_ESM.tiff]

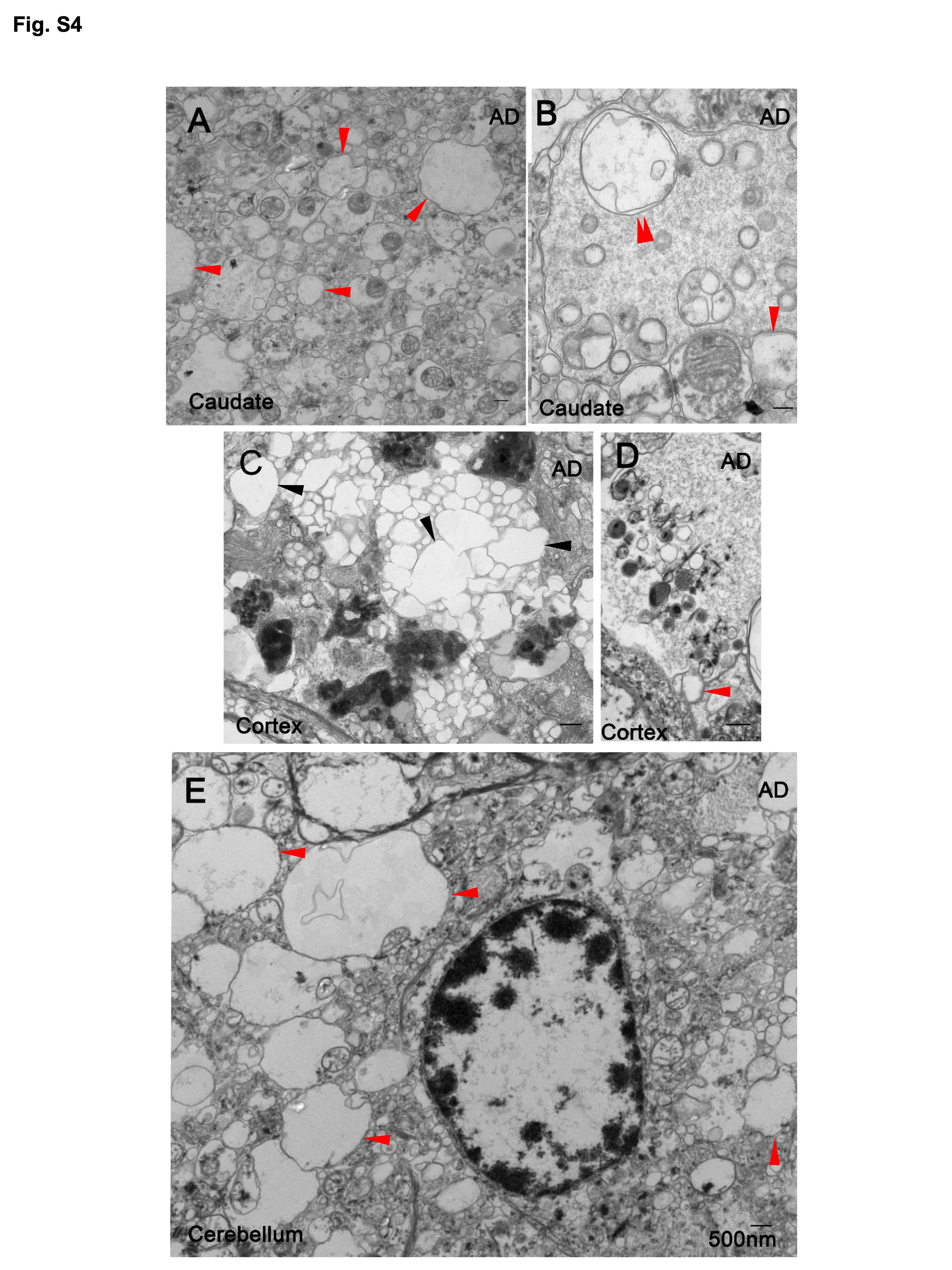

Supplement: Supplementary file 14 — Additional file 14. [file 40478_2025_2131_MOESM14_ESM.tiff]

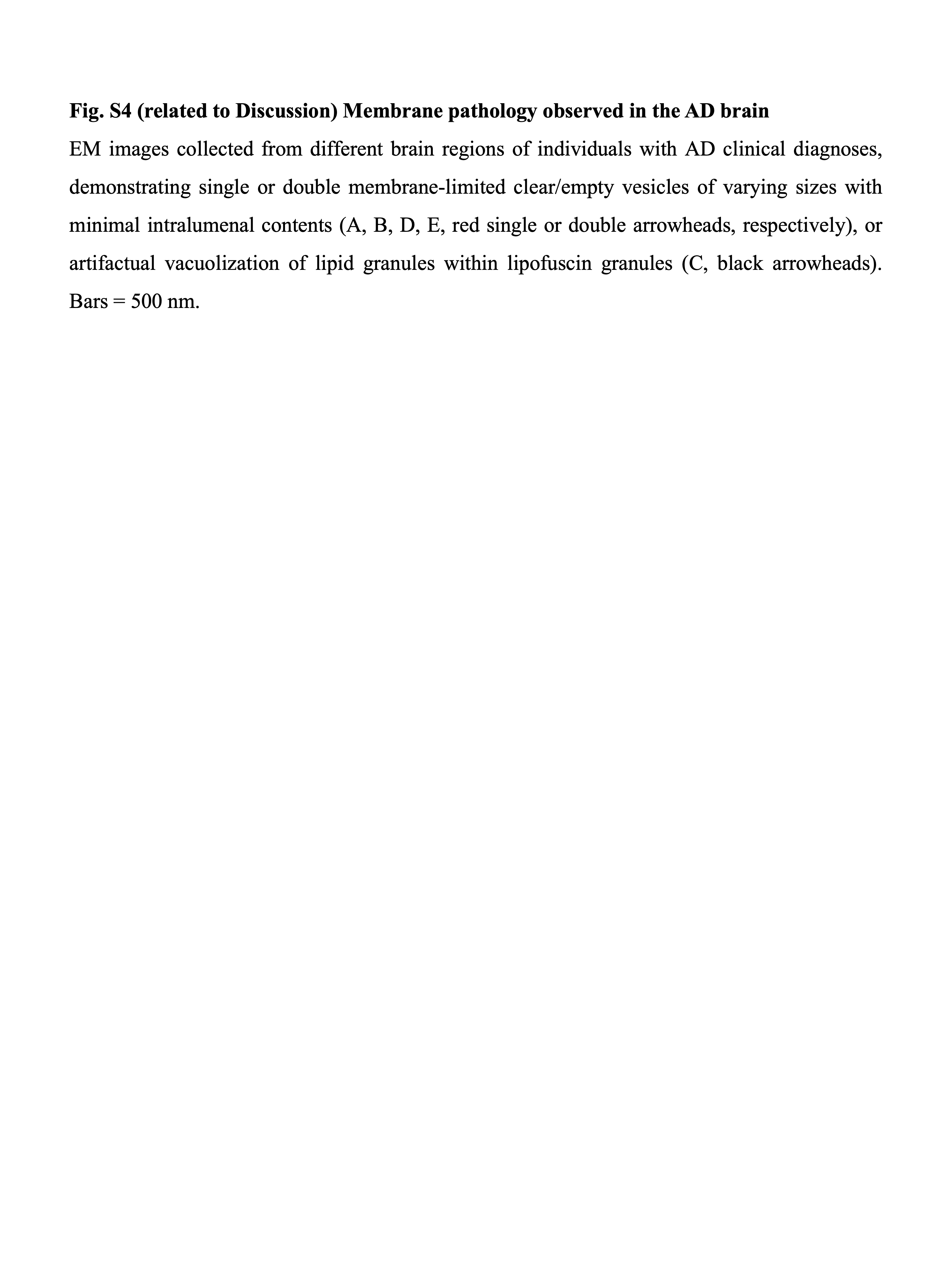

Supplement: Supplementary file 15 — Additional file 15. [file 40478_2025_2131_MOESM15_ESM.tiff]

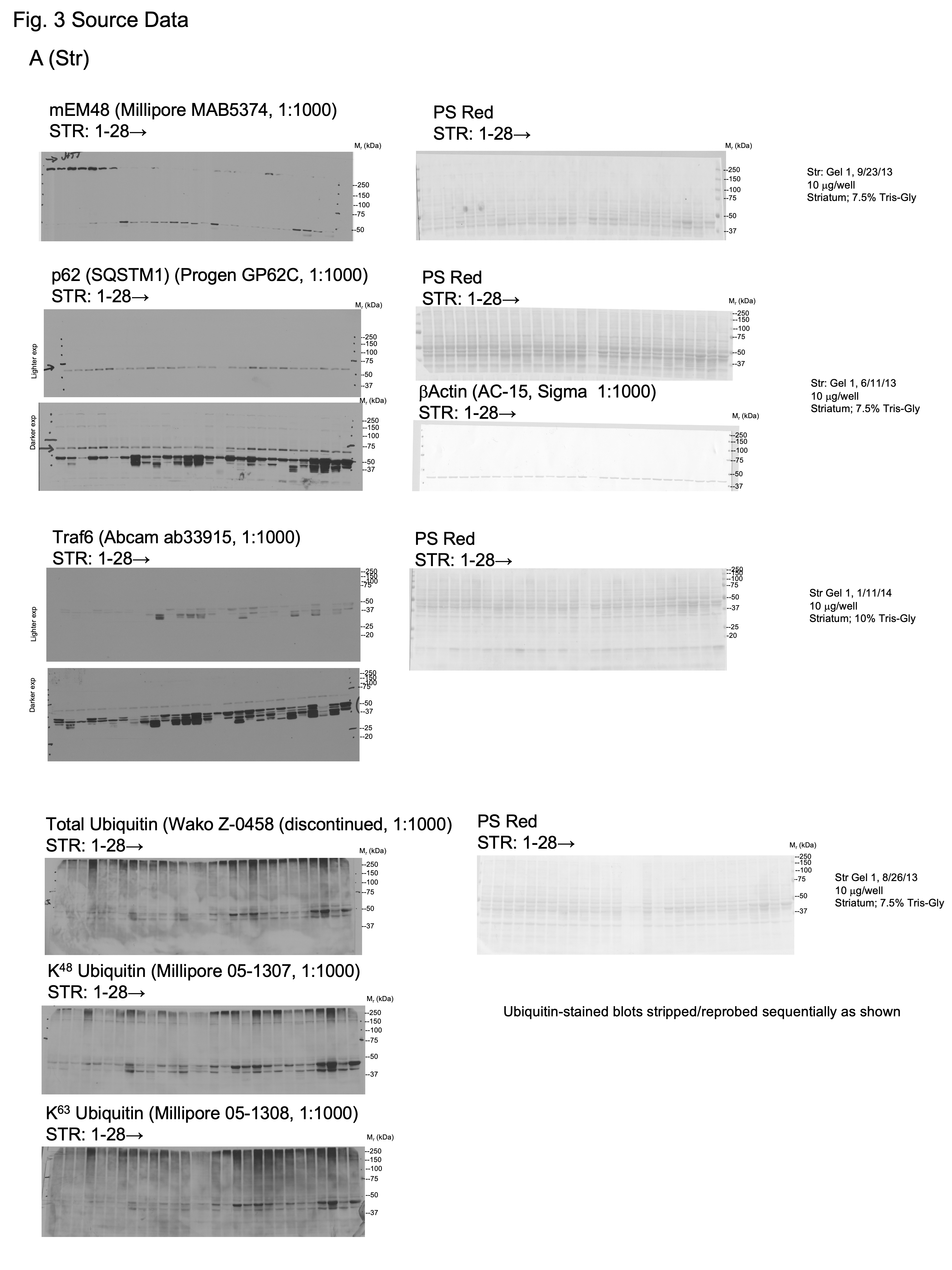

Supplement: Supplementary file 16 — Additional file 16. [file 40478_2025_2131_MOESM16_ESM.tiff]
